# Supplementary material for: Deciphering the molecular logic of WOX5 function in the root stem cell organizer
Source: EMBO J. 2024 Nov 18;44(1):281–303. doi: 10.1038/s44318-024-00302-2 (PMC11696986; doi:10.1038/s44318-024-00302-2)
Supplement: Supplementary file 1 — Appendix [file 44318_2024_302_MOESM1_ESM.pdf]

Appendix for

Deciphering the molecular logic of WOX5 function in the root stem cell organizer

Appendix Figure S1. Low-input transcriptome, histone modification, and chromatin accessibility omics data obtained through FANS……………………………………………………………………………………...........................P2

Appendix Figure S2. dHMs and dACRs in CC and QC……………………………………………………………..P3

Appendix Figure S3. RT-qPCR of *TAA1*, *HAN*, *CDF4*, and *CYCD3;3*……………………………………………P4

Appendix Figure S4. Venn diagrams of QC-up DEGs and WOX5 activated DEGs……...……………………...P5

Appendix Figure S5. WOX5 ChIP-Chip data analysis……………………………………………………………...P6

Appendix Figure S6. Genomic distributions of dHMs and dACRs……………………………………………...P7

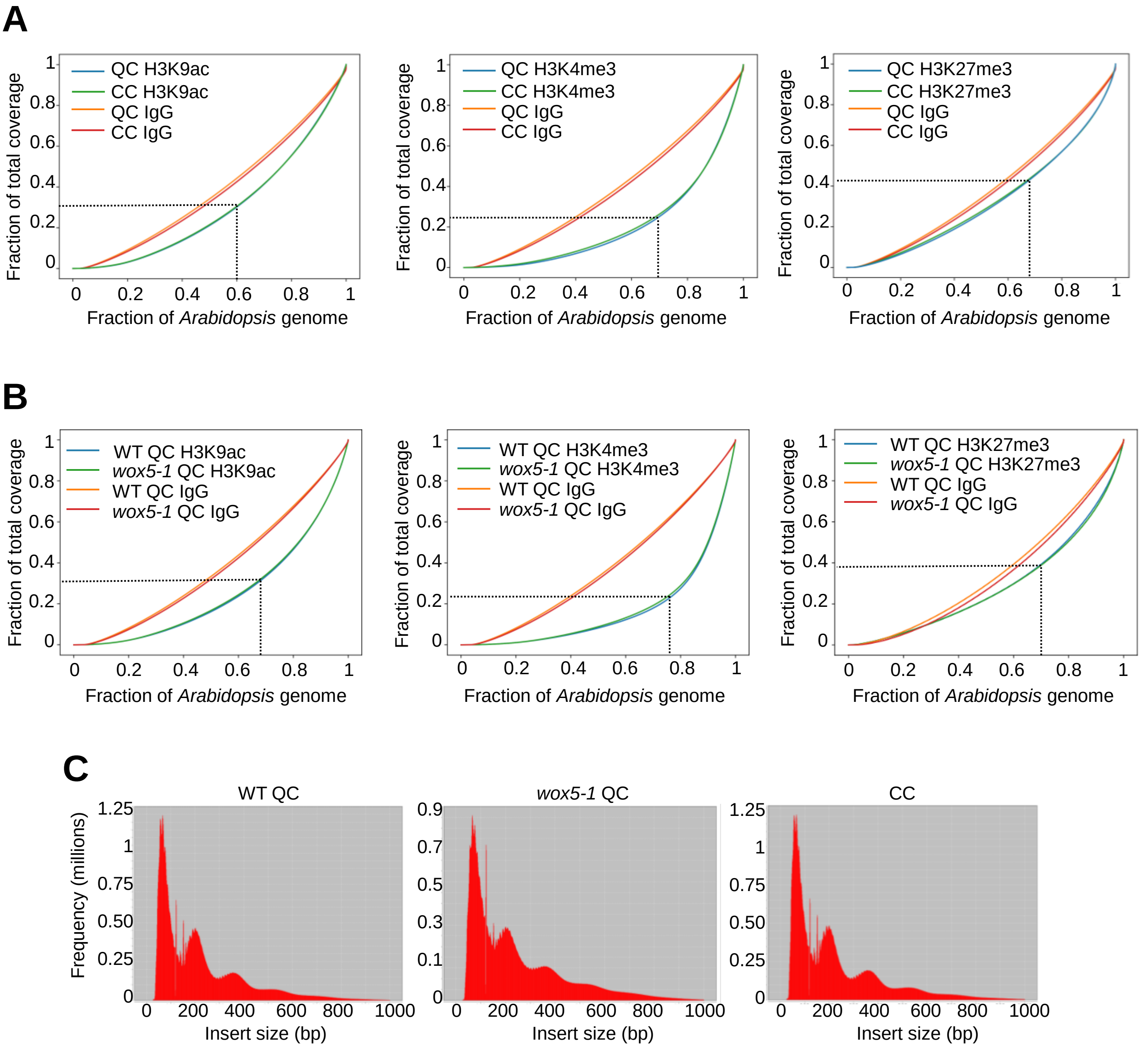

Appendix Figure S1. Low-input histone modification, and chromatin accessibility data obtained through FANS.

A-B Graphical summary of CUT&RUN peak enrichment for CC and QC from wild-type (**A**), and QC from wild-type (WT QC) and *wox5-1* (*wox5-1* QC) nuclei (**B**). The fingerprint plots of the indicated samples show the cumulative percentages of total reads (y-axis) against the fraction of the *A. thaliana* genome (x-axis). The intersection of dotted lines denotes the elbow point.

C ATAC fragment size distribution of the indicates samples. The peaks represent the mono-, di- and tri-nucleosome containing DNA fragments.

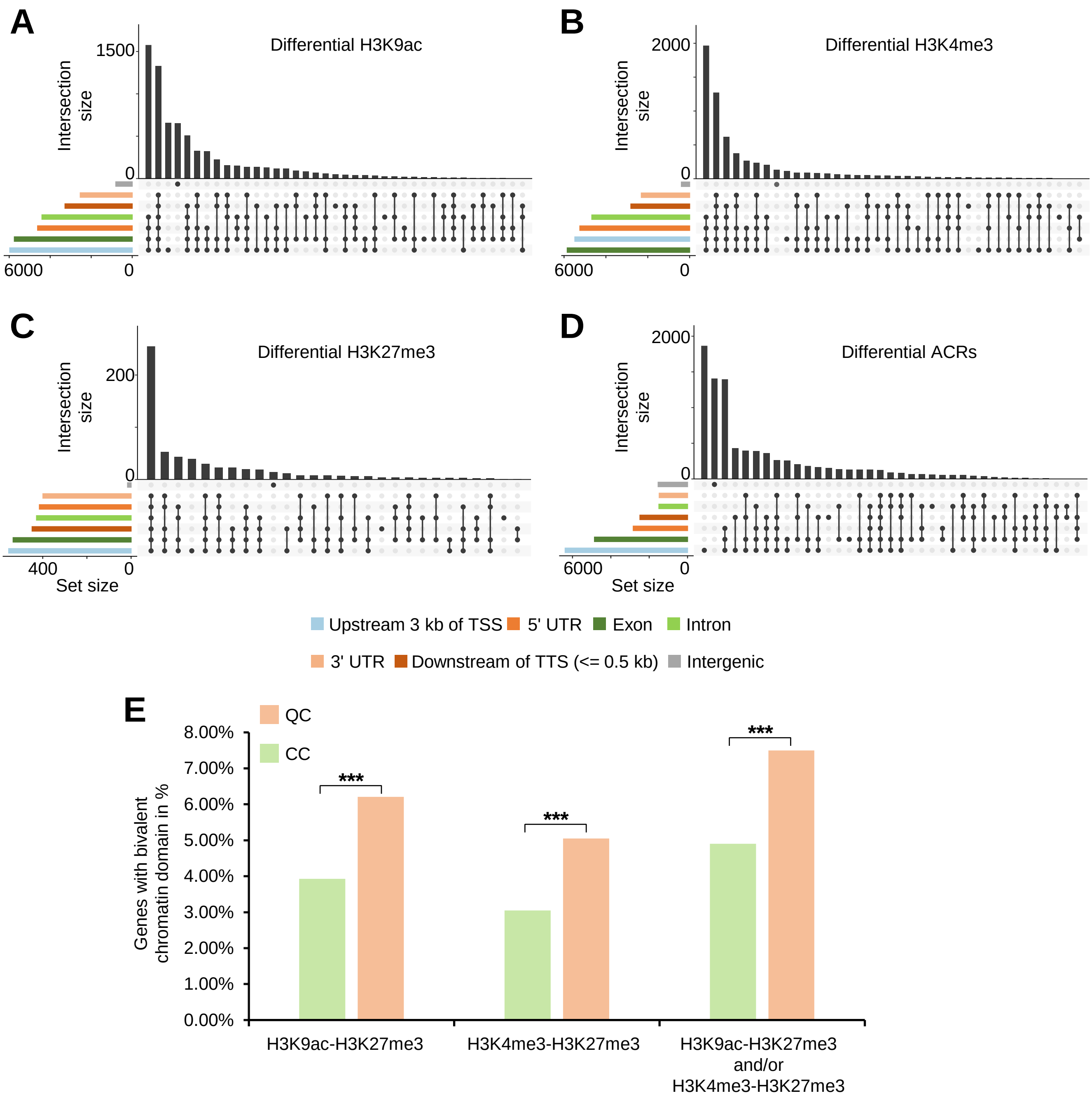

Appendix Figure S2. dHMs and dACRs in CCs and QC.

A-D Upset plots showing the genomic distributions of the indicated differential chromatin features in CCs versus QC (CC/QC). The vertical bars represent the numbers of the peaks spanning one or more genomic regions, with overlaps indicated by connection lines between the black dots. The horizontal bars indicate the total number of peaks for each genomic annotation, independent of overlaps.

E Percentage of genes with bivalent chromatin domains within 3 kb upstream of the TSS compared to all expressed genes in QC or CC. ***, $P < 0.001$; by Chi-Square tests.

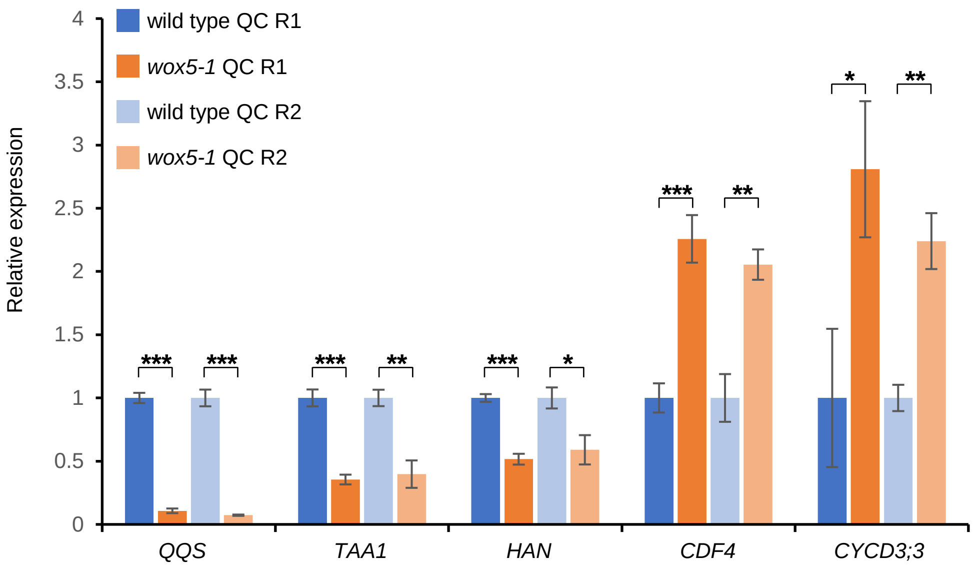

Appendix Figure S3. RT-qPCR of *TAA1*, *HAN*, *CDF4*, and *CYCD3;3.*

Relative transcript levels of *TAA1*, *HAN*, *CDF4*, and *CYCD3;3* measured by RT-qPCR from sorted nuclei of *wox5-1* and wild-type QC. The WOX5 activated DEG *QUA-QUINE STARCH* (*QQS*) from our data is used as a positive control. Error bars represent the SDs of three technical replicates. *, $P < 0.05$; **, $P < 0.01$; ***, $P < 0.001$; by two-sided Student's *t* test.

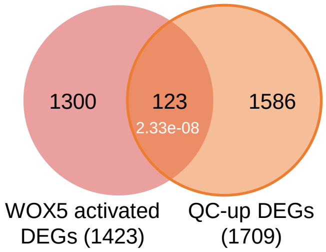

Appendix Figure S4. Venn diagrams of QC-up DEGs and WOX5-activated DEGs. The P value indicates the statistical significance of the overlap by hypergeometric test.

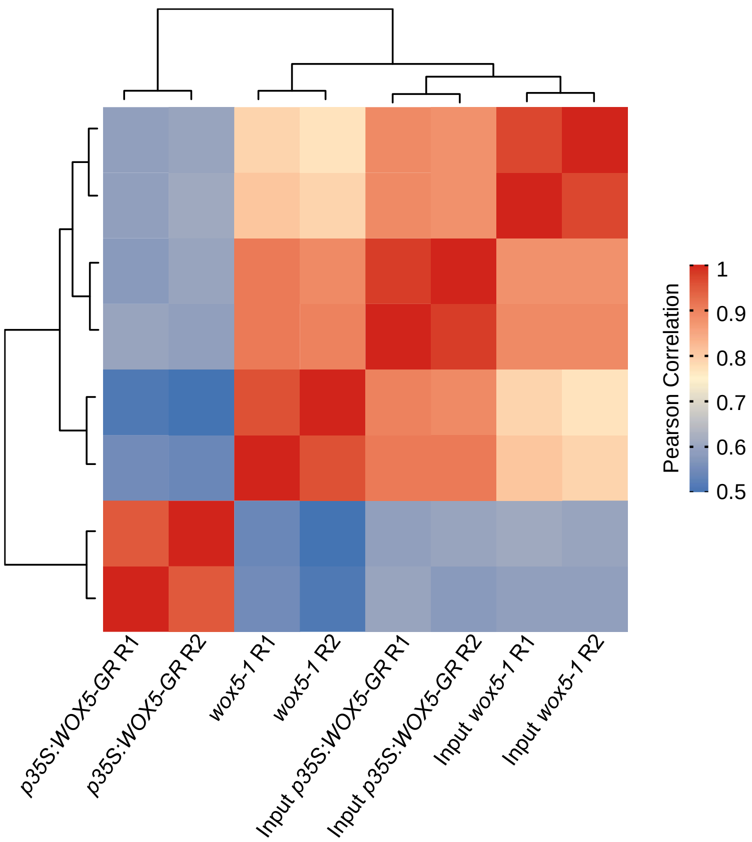

Appendix Figure S5. WOX5 ChIP-Chip data analysis.

Heatmap showing the Pearson correlation of biological replicates of the WOX5 ChIP-Chip experiments.

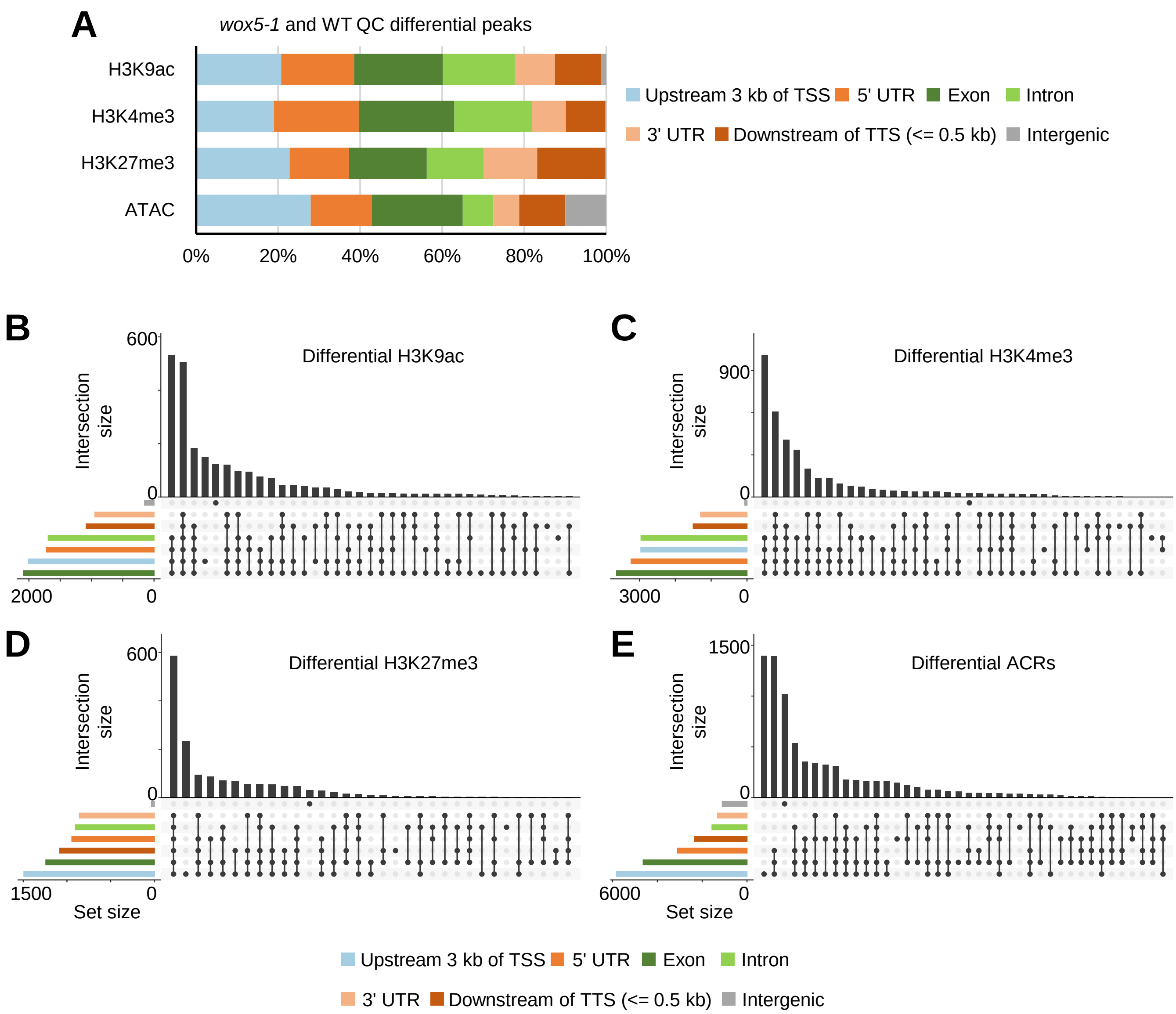

Appendix Figure S6. Genomic distributions of dHMs and dACRs.

A Genomic distributions of the indicated differential chromatin features between *wox5-1* and wild-type QC nuclei.

B-E In the Upset plots, the vertical bars represent the numbers of the peaks spanning one or more specific genomic regions, with overlaps indicated by connection lines between the black dots. The horizontal bars indicate the total number of peaks for each genomic annotation, independent of overlaps.
