# Supplementary figures and images for: Deciphering the molecular logic of WOX5 function in the root stem cell organizer
Source: EMBO J. 2024 Nov 18;44(1):281–303. doi: 10.1038/s44318-024-00302-2 (PMC11696986; doi:10.1038/s44318-024-00302-2)

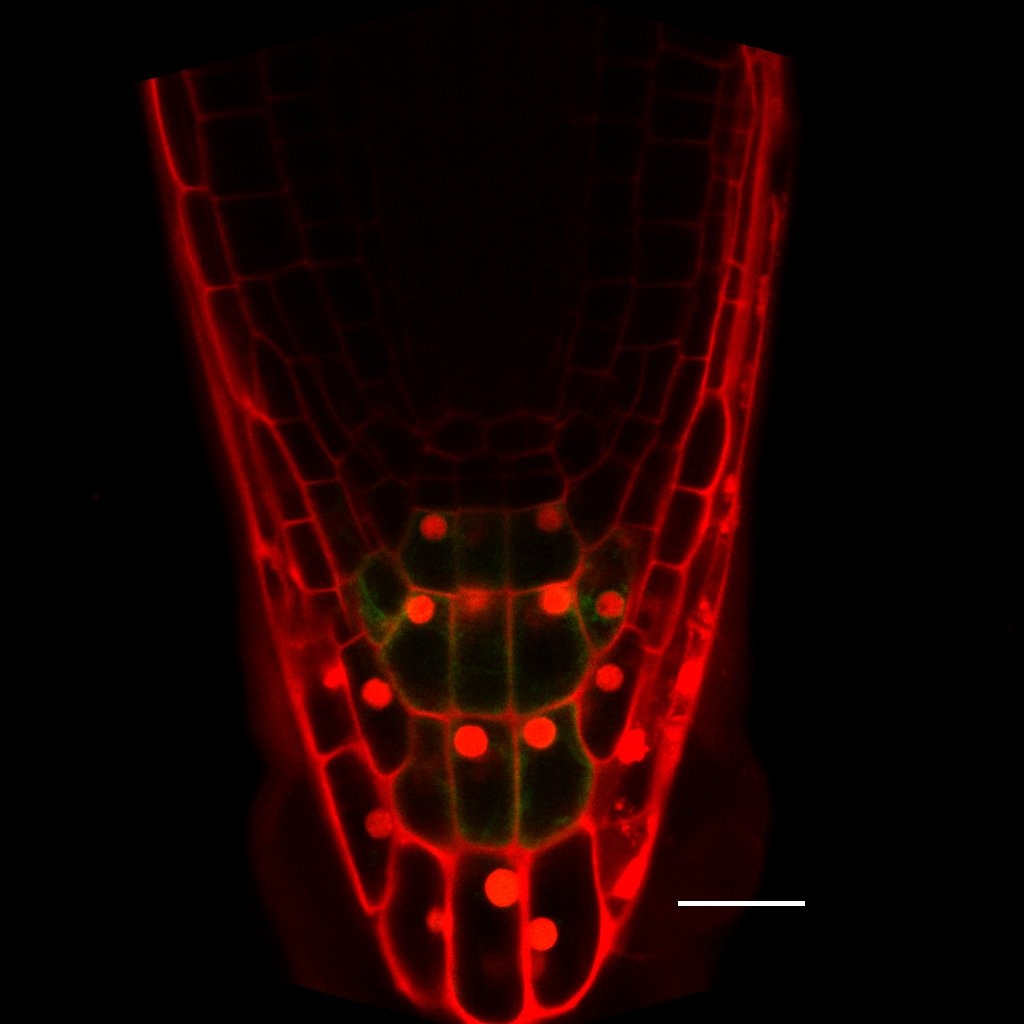

Supplement: Supplementary file 19 — Source data Fig. 1 [file 44318_2024_302_MOESM19_ESM.zip › Figure 1/1B/CC.tiff]

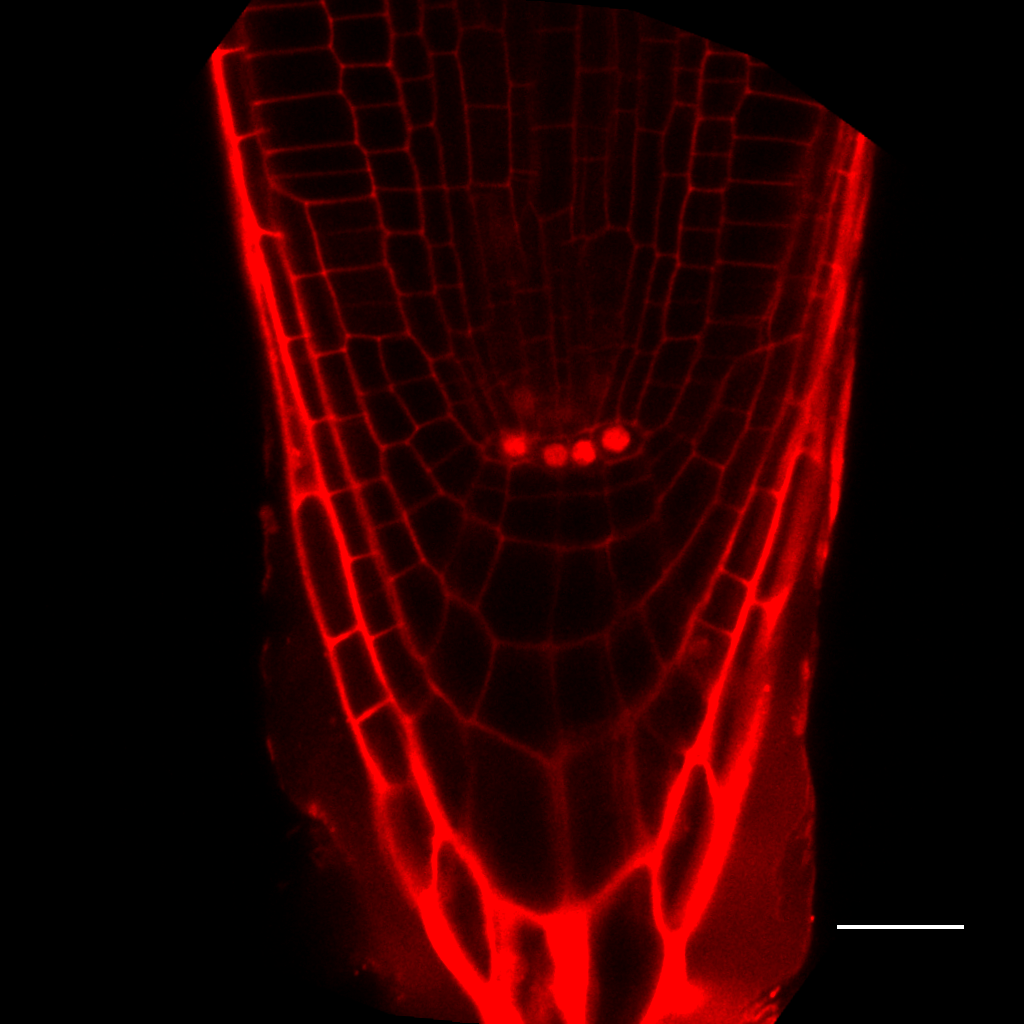

Supplement: Supplementary file 19 — Source data Fig. 1 [file 44318_2024_302_MOESM19_ESM.zip › Figure 1/1B/WT_QC.tiff]

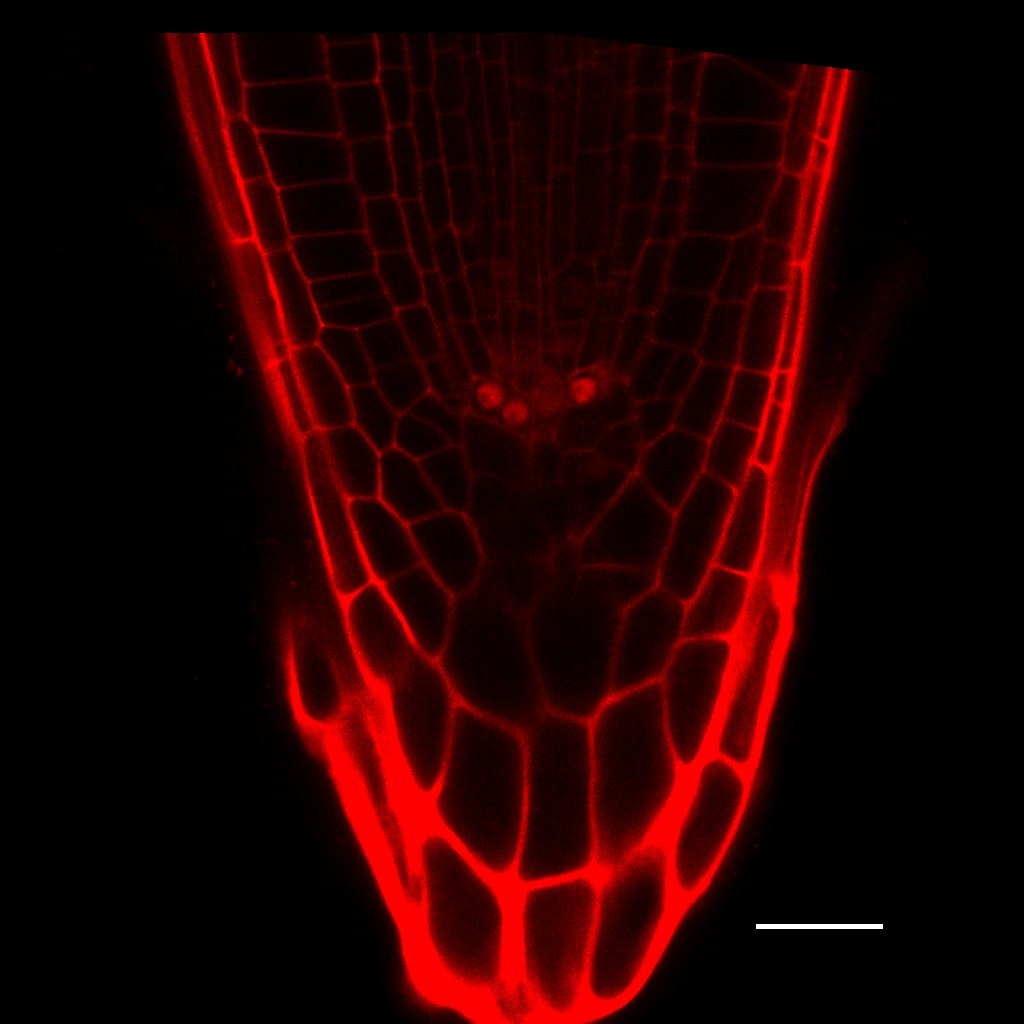

Supplement: Supplementary file 19 — Source data Fig. 1 [file 44318_2024_302_MOESM19_ESM.zip › Figure 1/1B/wox5-1_QC.tiff]

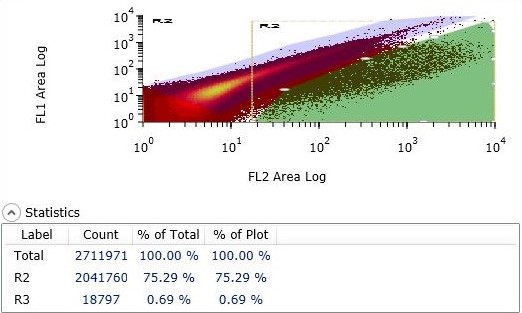

Supplement: Supplementary file 19 — Source data Fig. 1 [file 44318_2024_302_MOESM19_ESM.zip › Figure 1/1C/CC tdTomato.tiff]

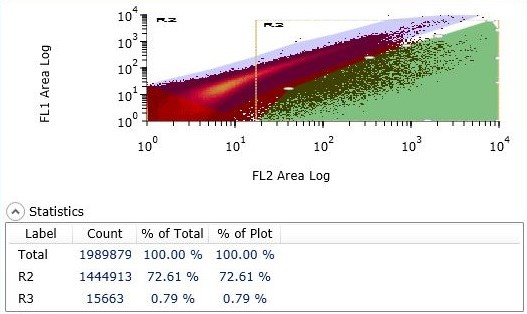

Supplement: Supplementary file 19 — Source data Fig. 1 [file 44318_2024_302_MOESM19_ESM.zip › Figure 1/1C/WT QC tdTomato.tiff]

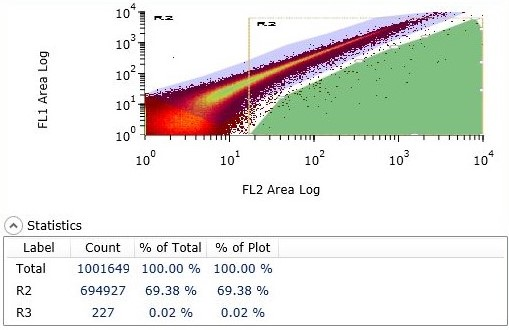

Supplement: Supplementary file 19 — Source data Fig. 1 [file 44318_2024_302_MOESM19_ESM.zip › Figure 1/1C/WT no fluorophore.tiff]

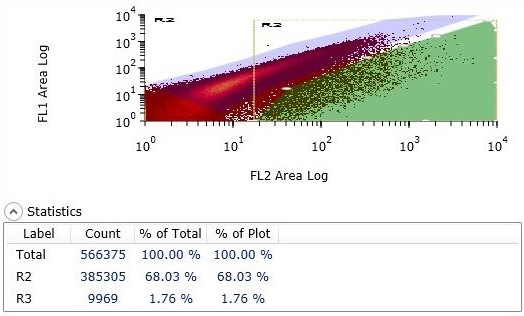

Supplement: Supplementary file 19 — Source data Fig. 1 [file 44318_2024_302_MOESM19_ESM.zip › Figure 1/1C/wox5-1 QC tdTomato.tiff]

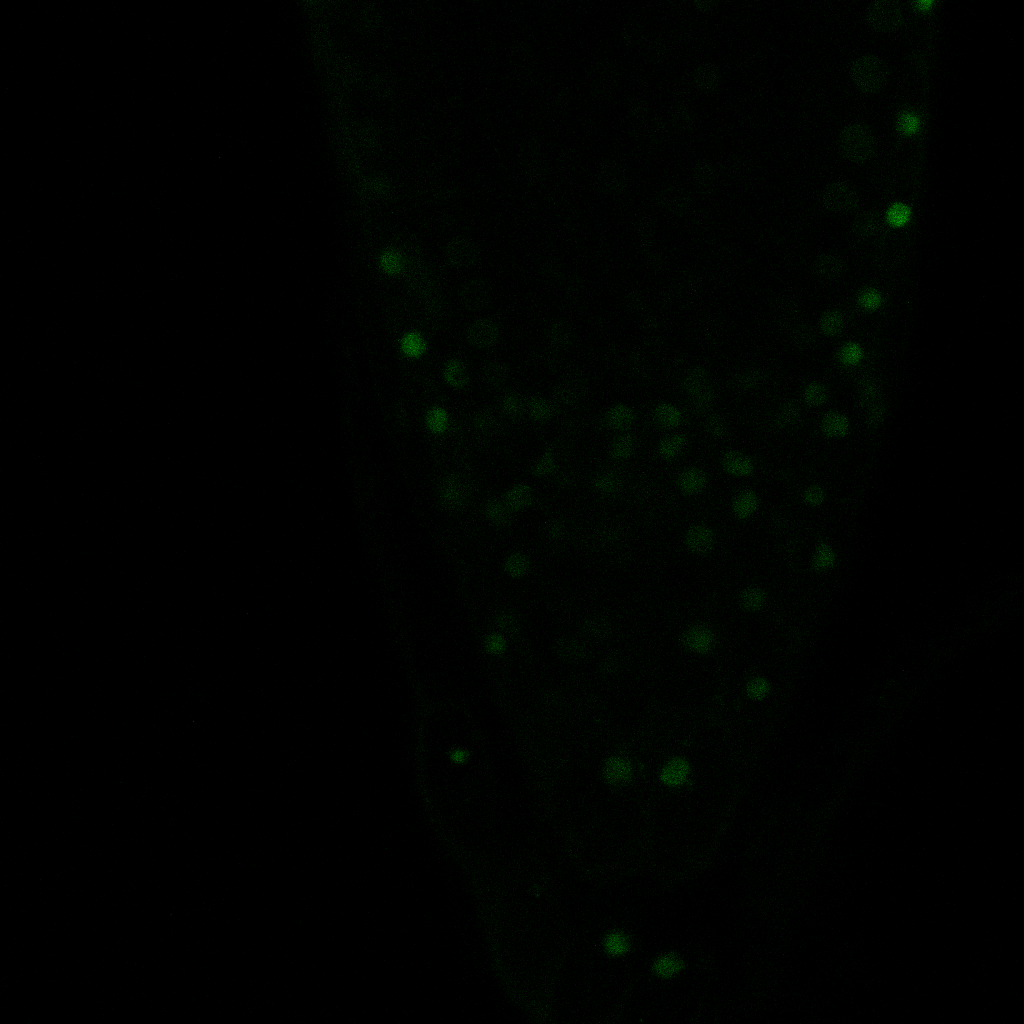

Supplement: Supplementary file 22 — Source data Fig. 5 [file 44318_2024_302_MOESM22_ESM.zip › Figure 5/5A/CESA1 in WT_Image GFP.tif]

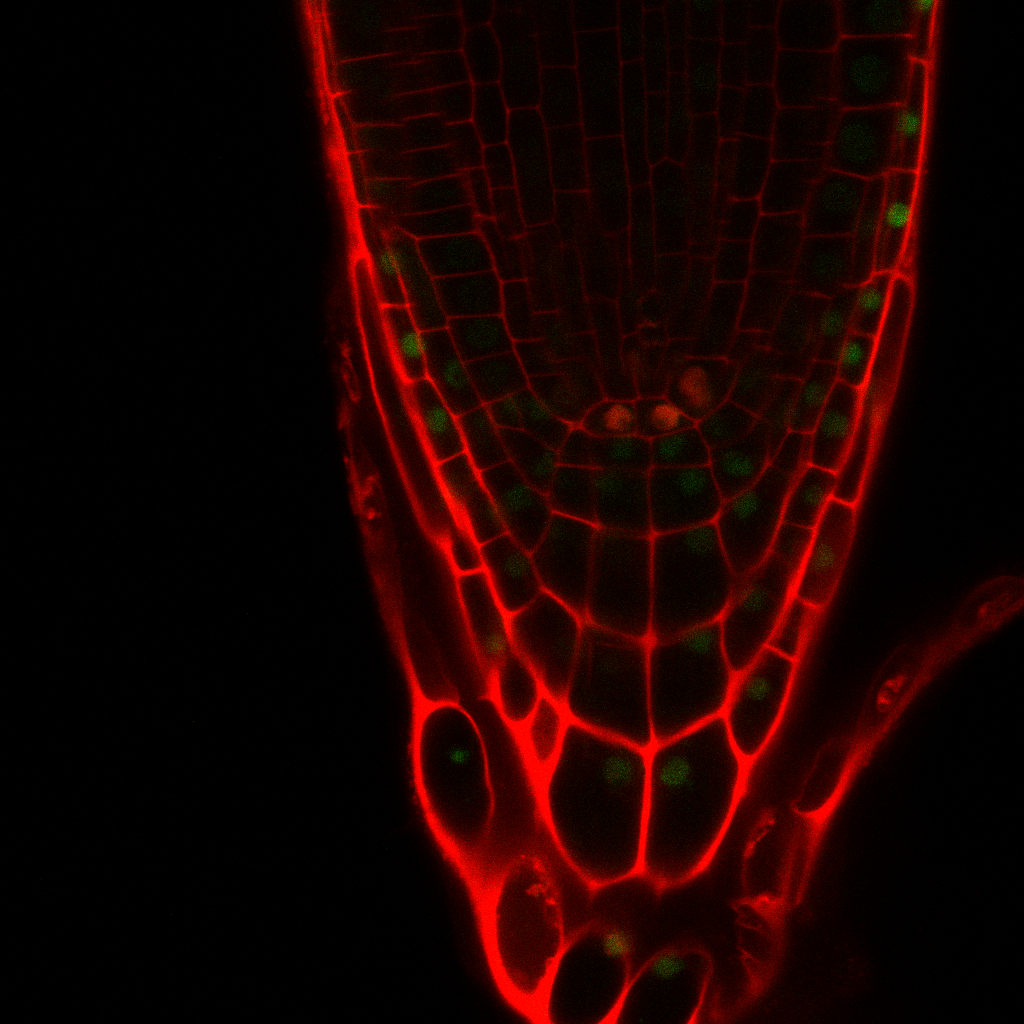

Supplement: Supplementary file 22 — Source data Fig. 5 [file 44318_2024_302_MOESM22_ESM.zip › Figure 5/5A/CESA1 in WT_Image tdTomato and GFP.tif]

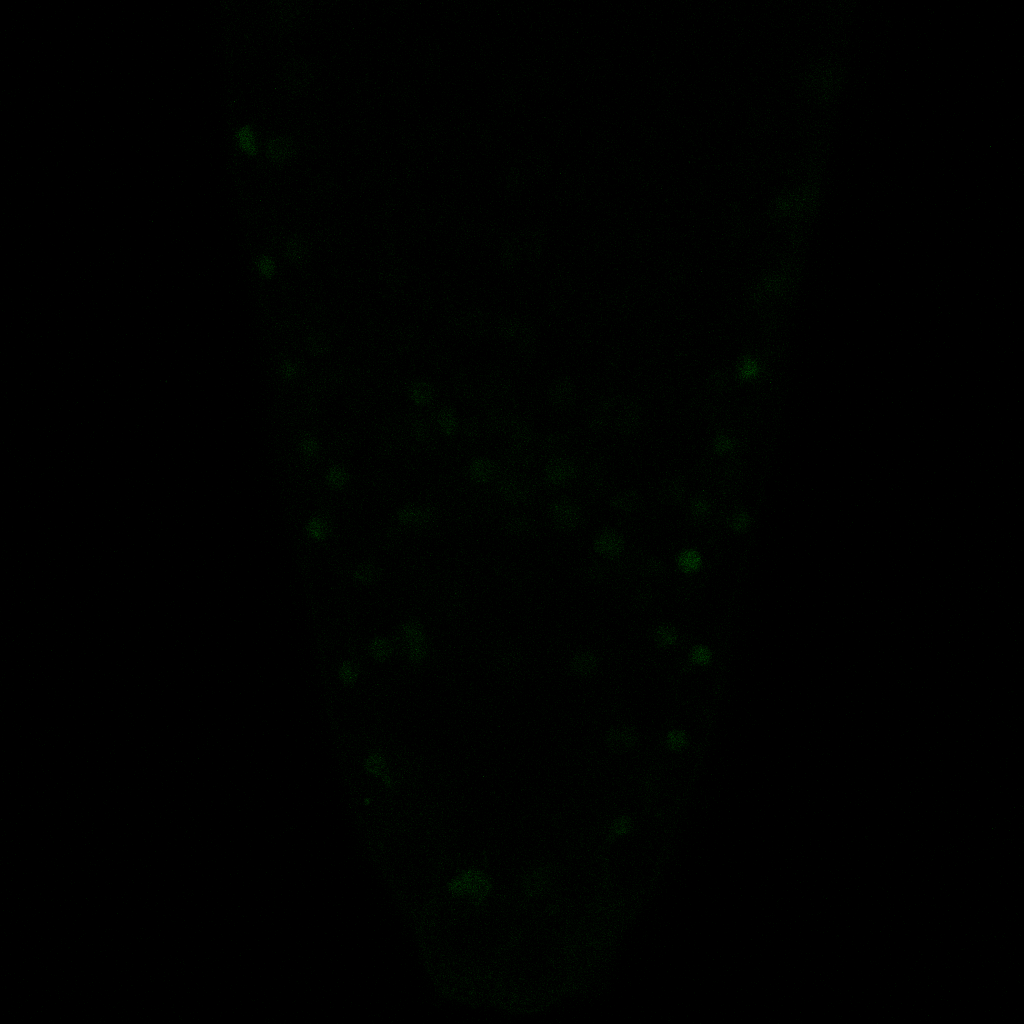

Supplement: Supplementary file 22 — Source data Fig. 5 [file 44318_2024_302_MOESM22_ESM.zip › Figure 5/5A/CESA1 in wox5-1_Image GFP.tif]

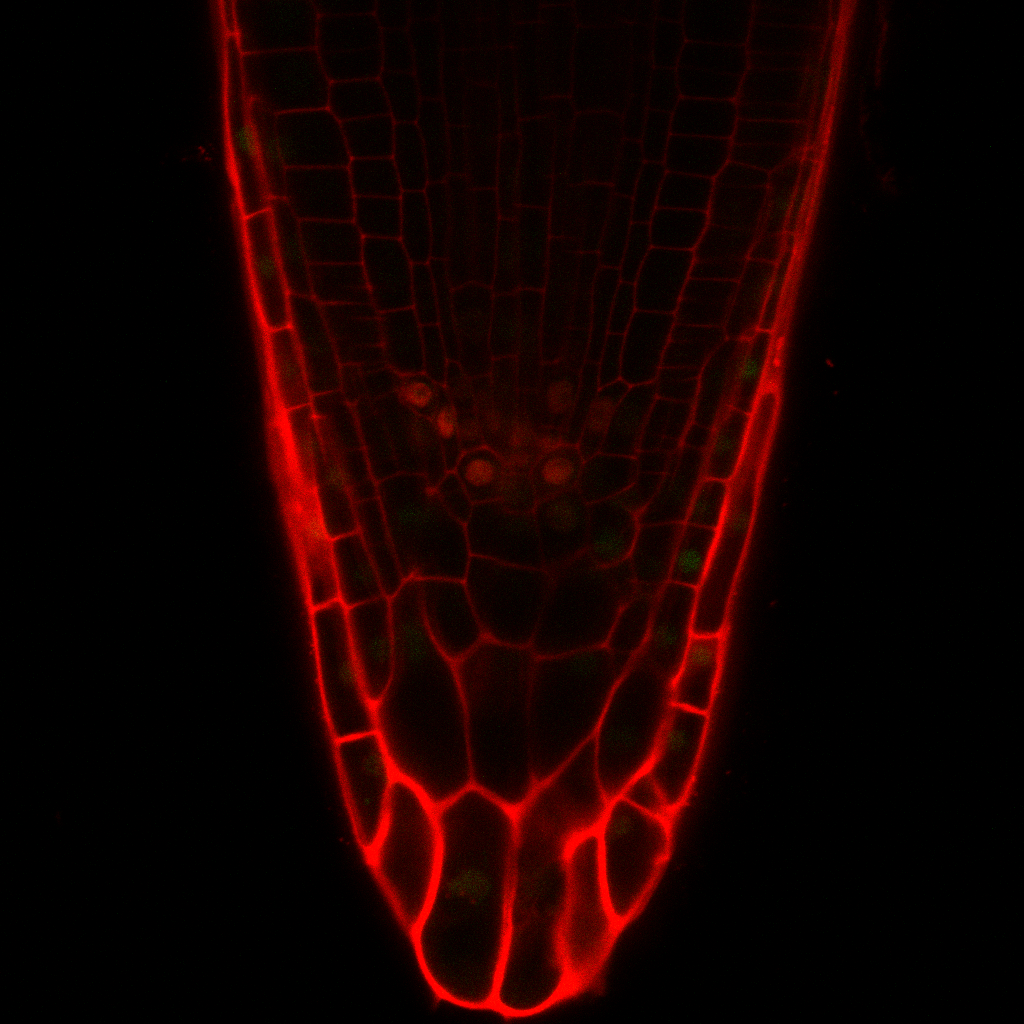

Supplement: Supplementary file 22 — Source data Fig. 5 [file 44318_2024_302_MOESM22_ESM.zip › Figure 5/5A/CESA1 in wox5-1_Image tdTomato and GFP.tif]

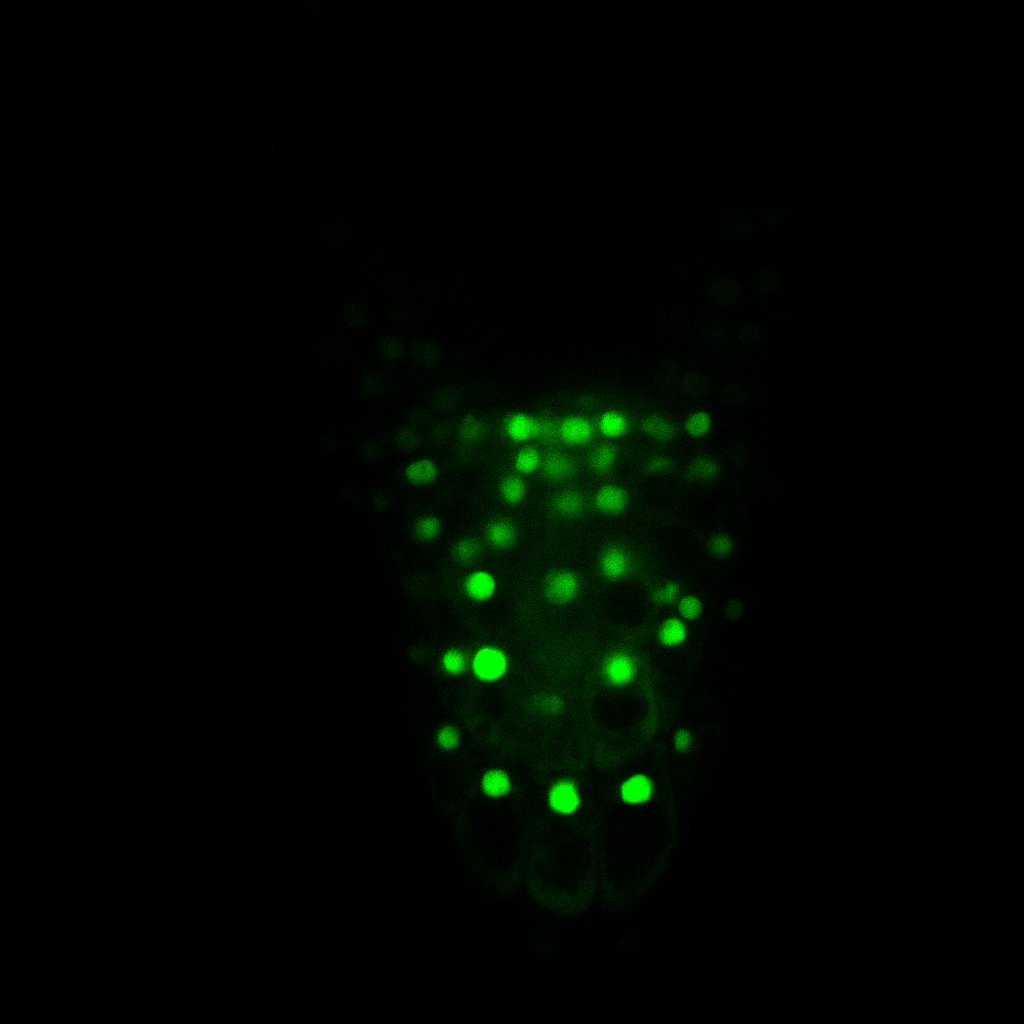

Supplement: Supplementary file 22 — Source data Fig. 5 [file 44318_2024_302_MOESM22_ESM.zip › Figure 5/5B/CEPR2 in WT_Image GFP.tif]

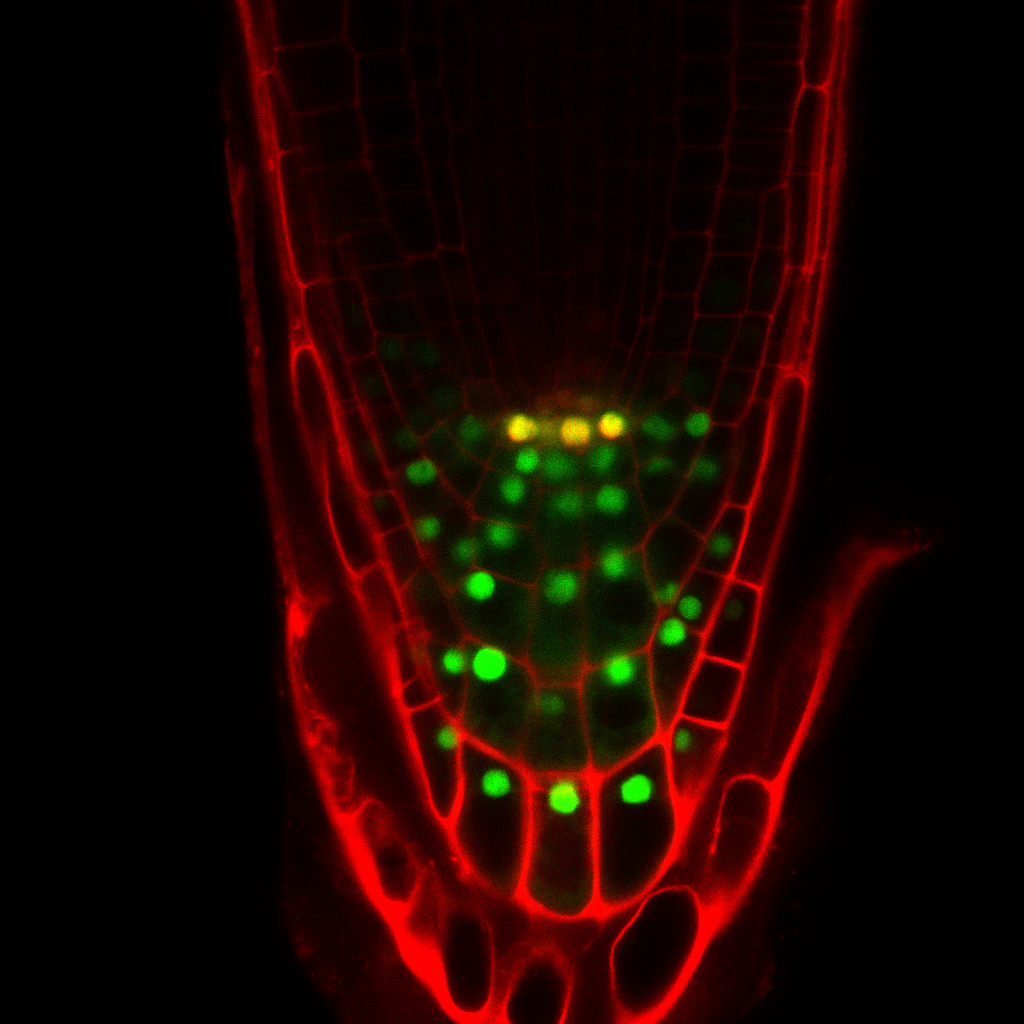

Supplement: Supplementary file 22 — Source data Fig. 5 [file 44318_2024_302_MOESM22_ESM.zip › Figure 5/5B/CEPR2 in WT_Image tdTomato and GFP.tif]

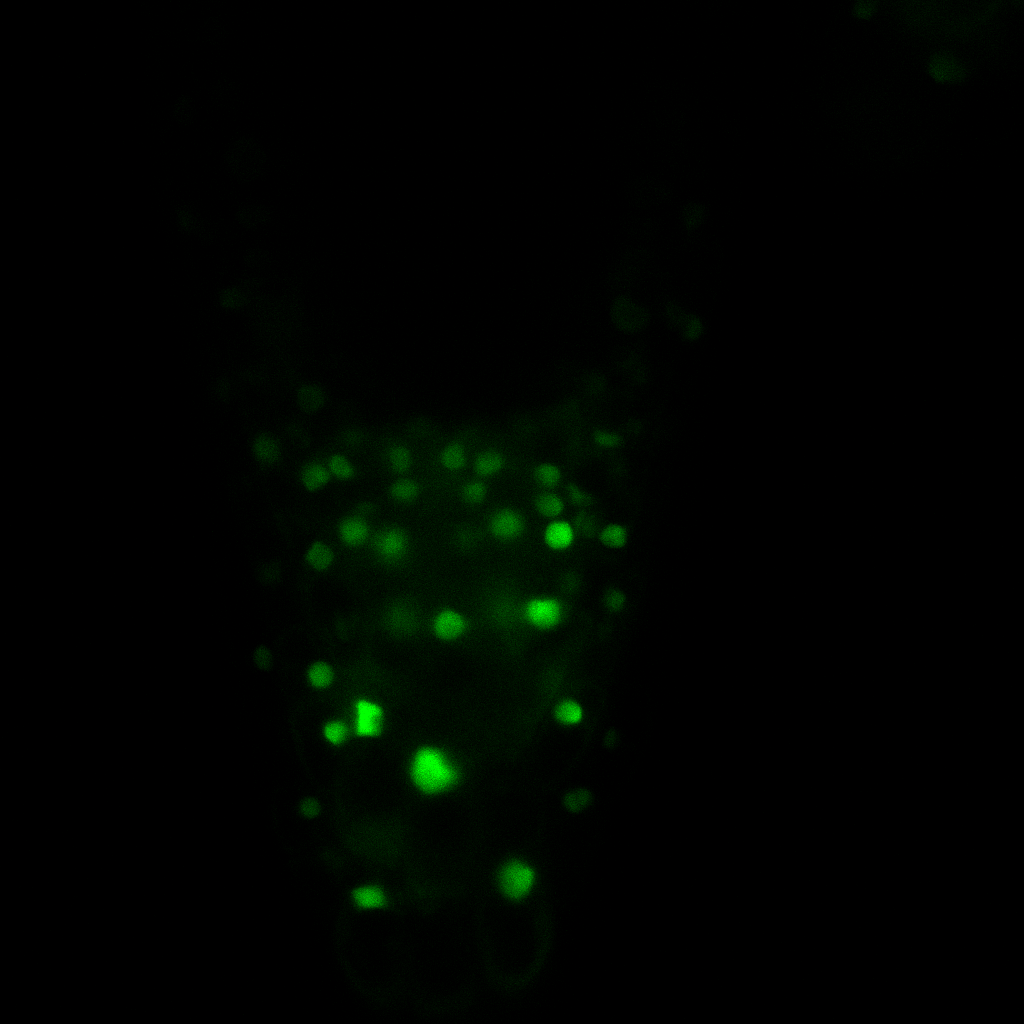

Supplement: Supplementary file 22 — Source data Fig. 5 [file 44318_2024_302_MOESM22_ESM.zip › Figure 5/5B/CEPR2 in wox5-1_Image GFP.tif]

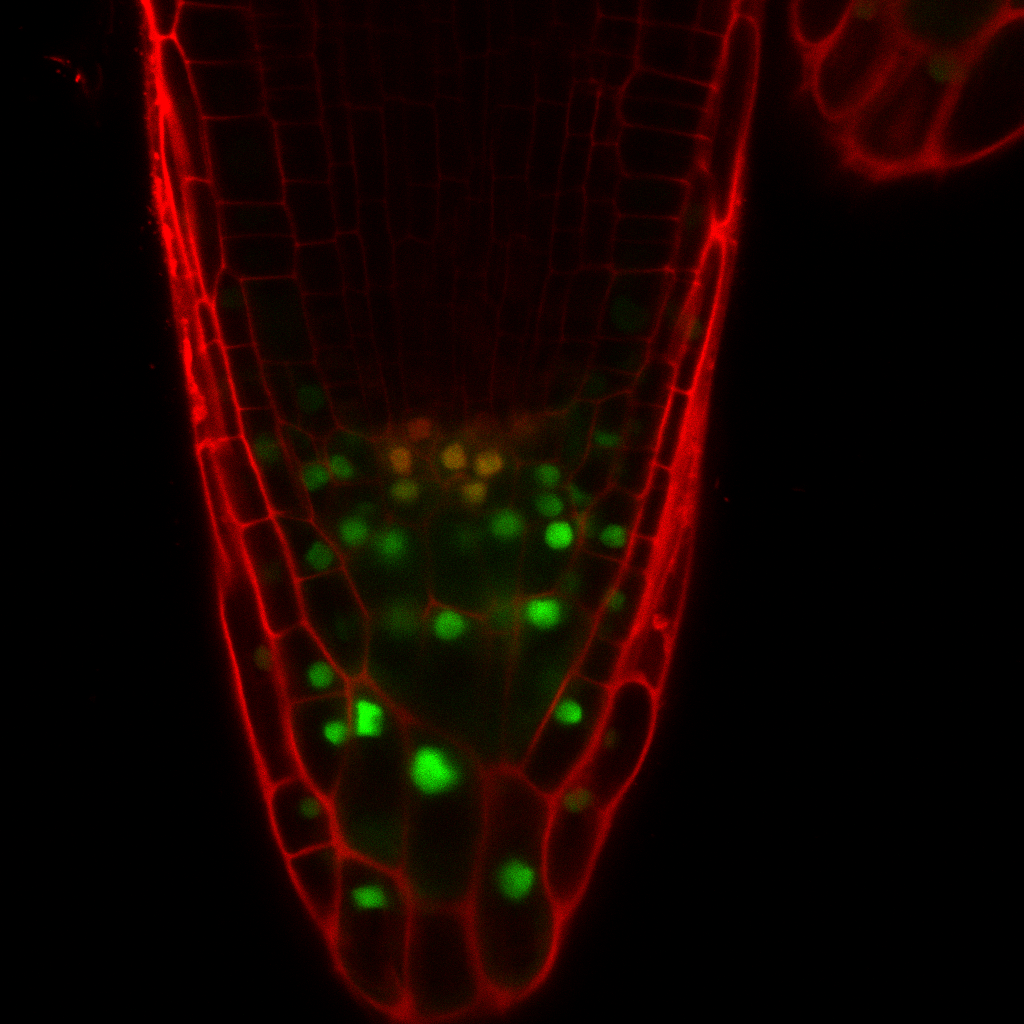

Supplement: Supplementary file 22 — Source data Fig. 5 [file 44318_2024_302_MOESM22_ESM.zip › Figure 5/5B/CEPR2 in wox5-1_Image tdTomato and GFP.tif]

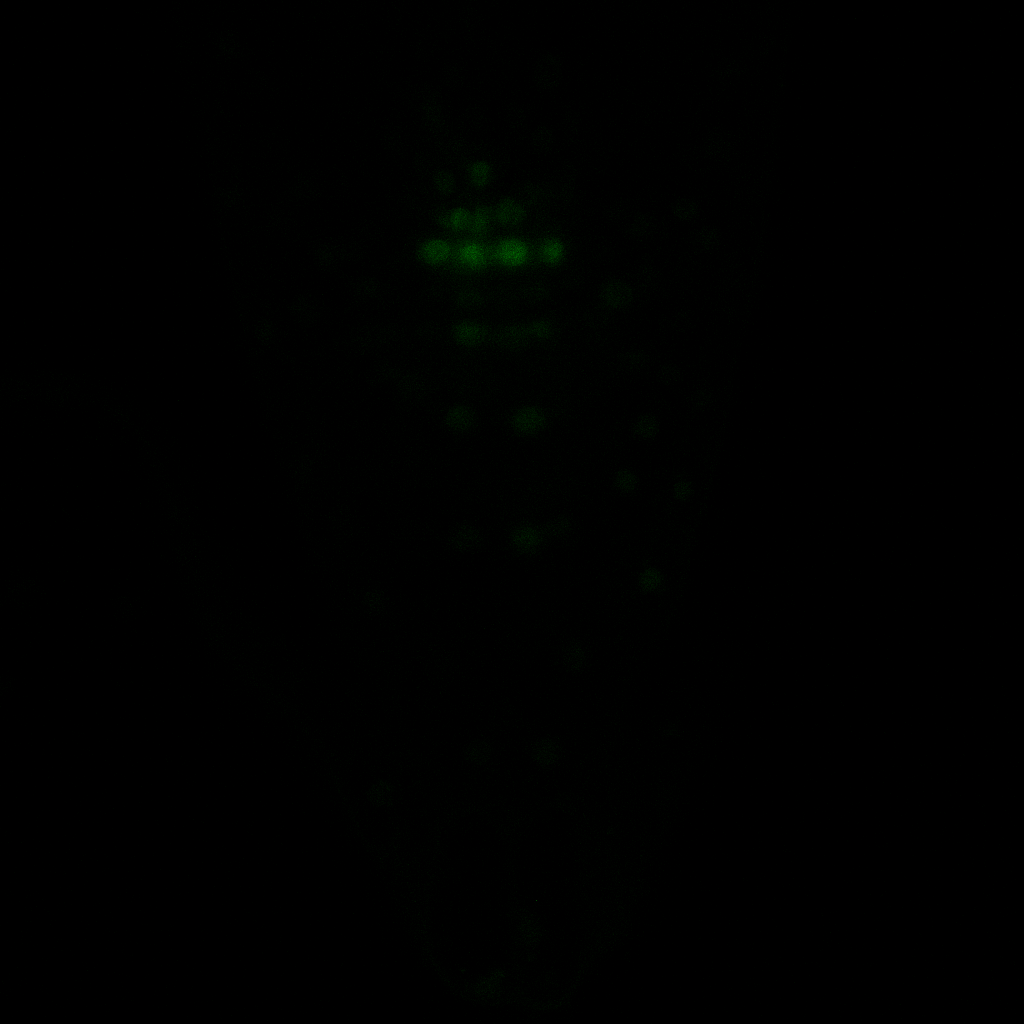

Supplement: Supplementary file 22 — Source data Fig. 5 [file 44318_2024_302_MOESM22_ESM.zip › Figure 5/5C/ERD14 in WT_Image GFP.tif]

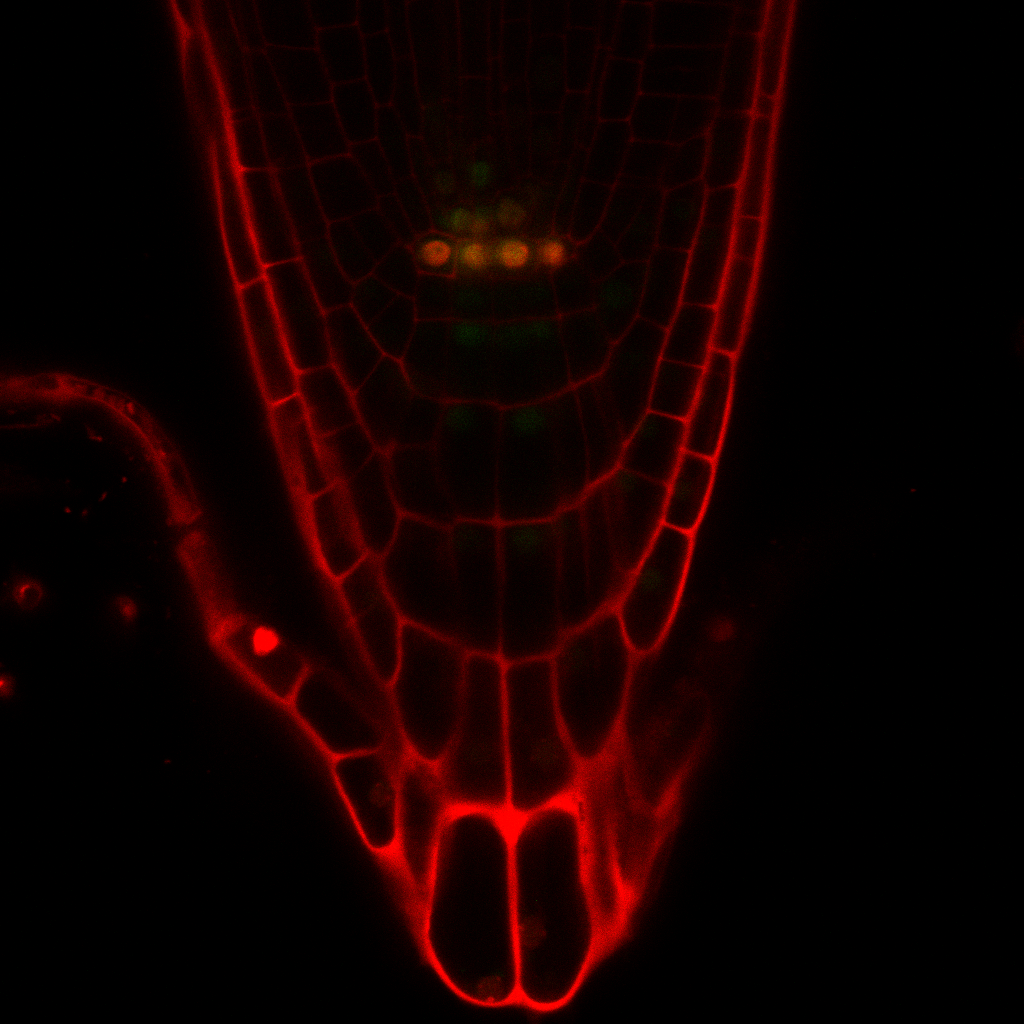

Supplement: Supplementary file 22 — Source data Fig. 5 [file 44318_2024_302_MOESM22_ESM.zip › Figure 5/5C/ERD14 in WT_Image tdTomato and GFP.tif]

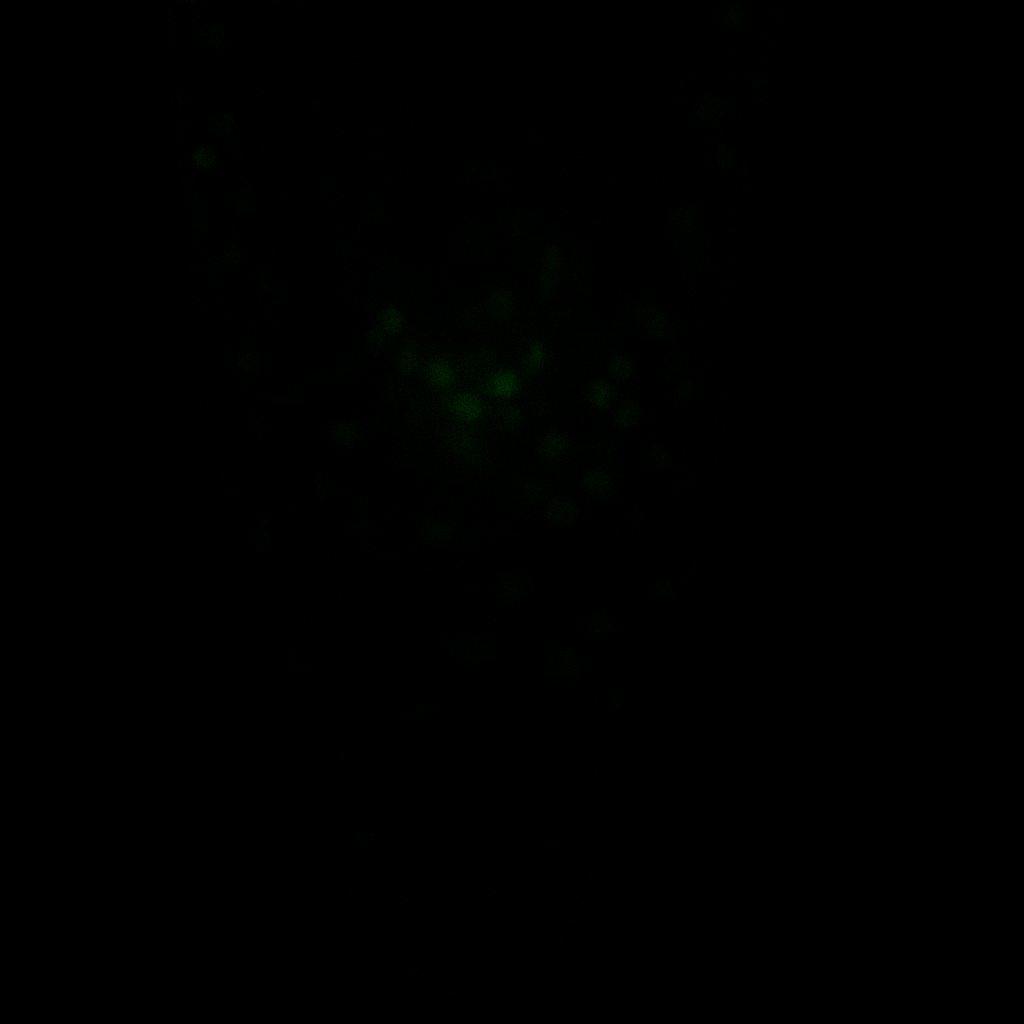

Supplement: Supplementary file 22 — Source data Fig. 5 [file 44318_2024_302_MOESM22_ESM.zip › Figure 5/5C/ERD14 in wox5-1_Image GFP.tif]

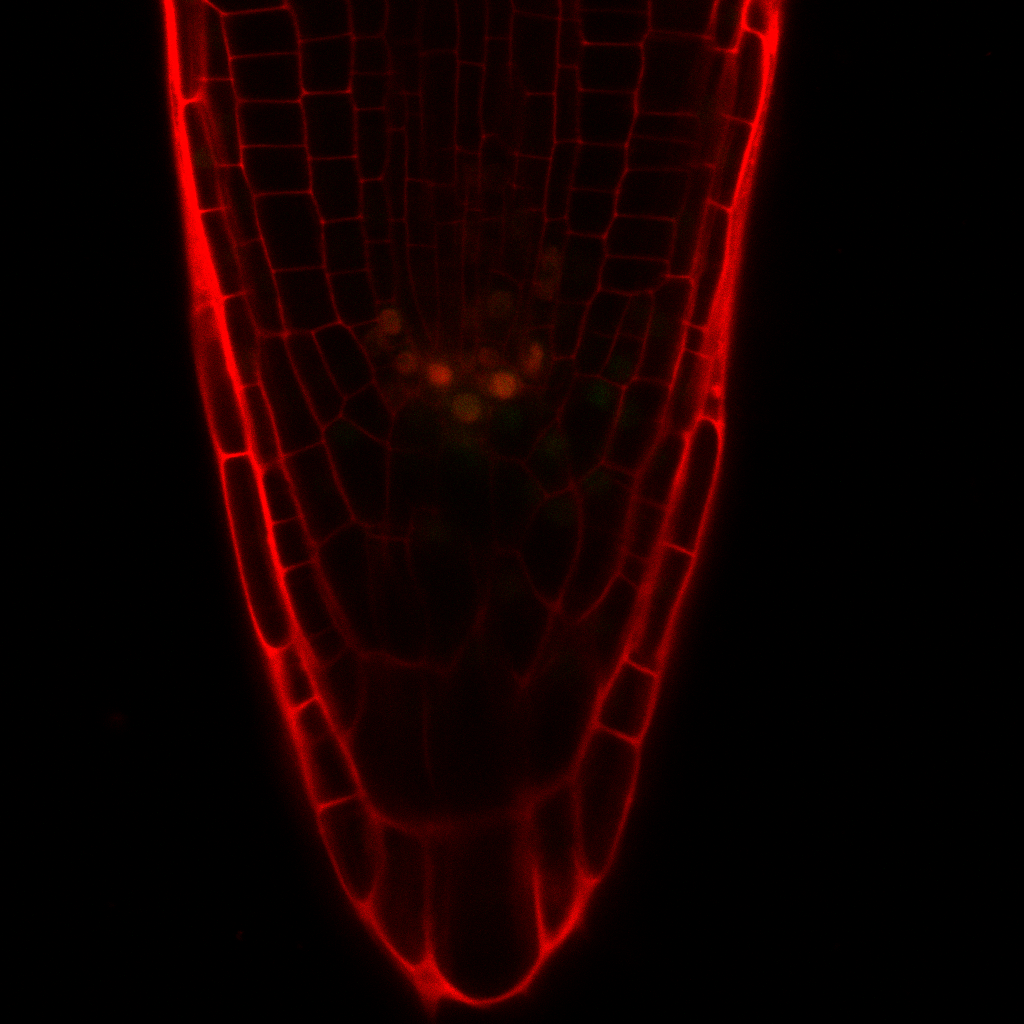

Supplement: Supplementary file 22 — Source data Fig. 5 [file 44318_2024_302_MOESM22_ESM.zip › Figure 5/5C/ERD14 in wox5-1_Image tdTomato and GFP.tif]

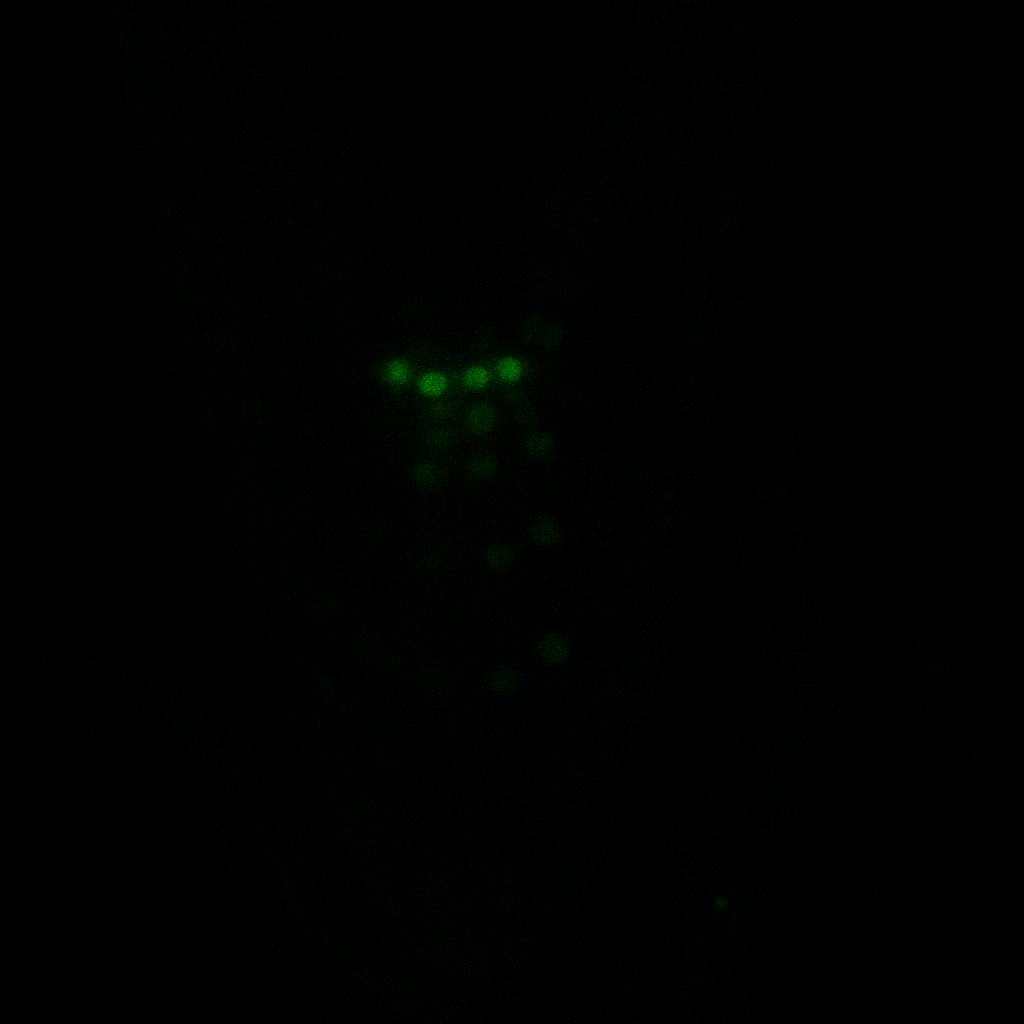

Supplement: Supplementary file 22 — Source data Fig. 5 [file 44318_2024_302_MOESM22_ESM.zip › Figure 5/5D/SGP1 in WT_Image GFP.tif]

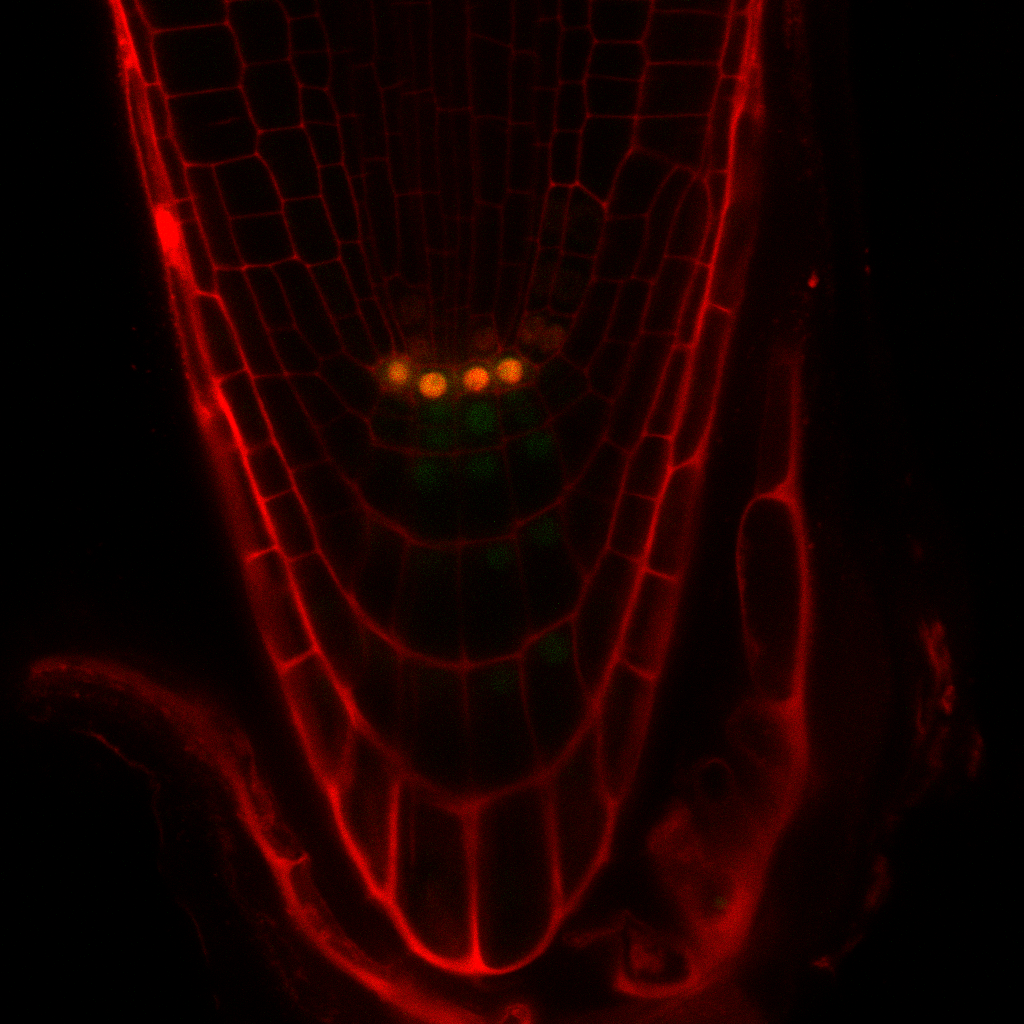

Supplement: Supplementary file 22 — Source data Fig. 5 [file 44318_2024_302_MOESM22_ESM.zip › Figure 5/5D/SGP1 in WT_Image tdTomato and GFP.tif]

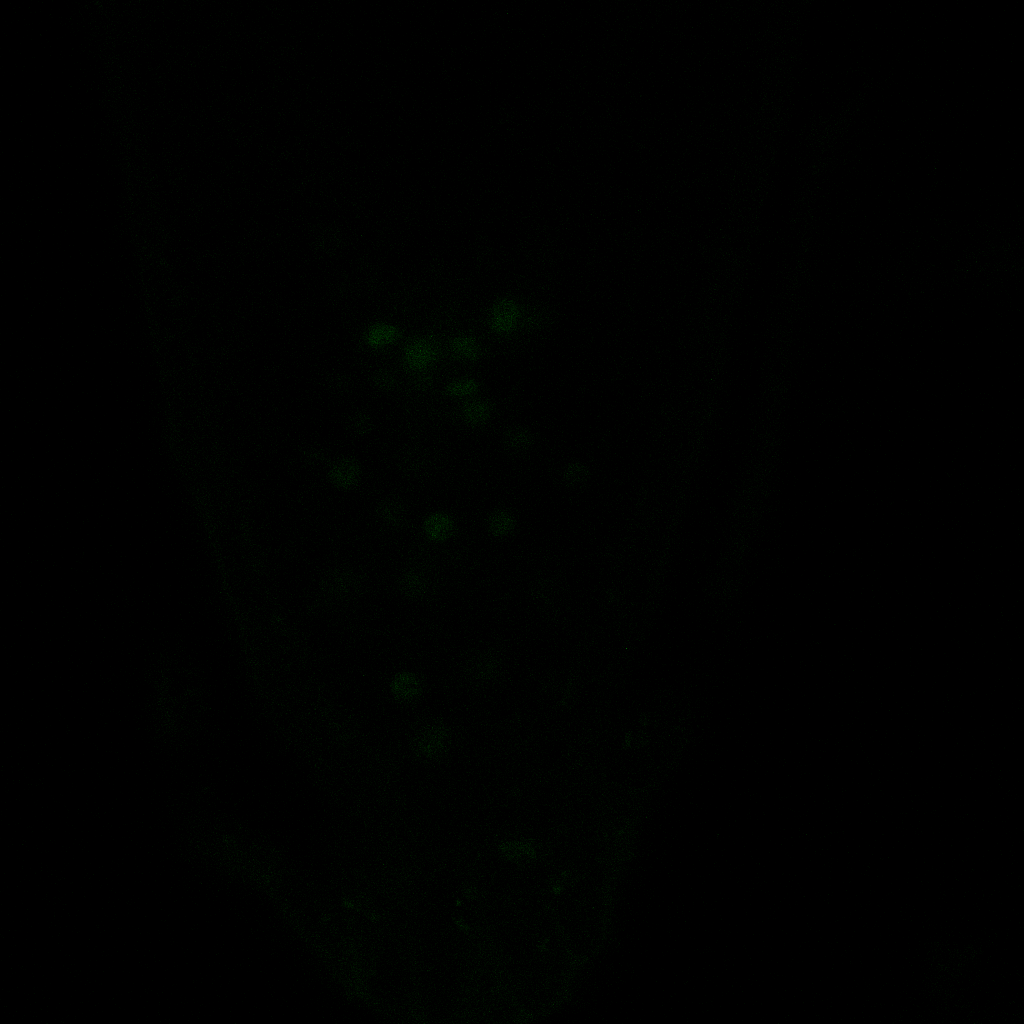

Supplement: Supplementary file 22 — Source data Fig. 5 [file 44318_2024_302_MOESM22_ESM.zip › Figure 5/5D/SGP1 in wox5-1_Image GFP.tif]

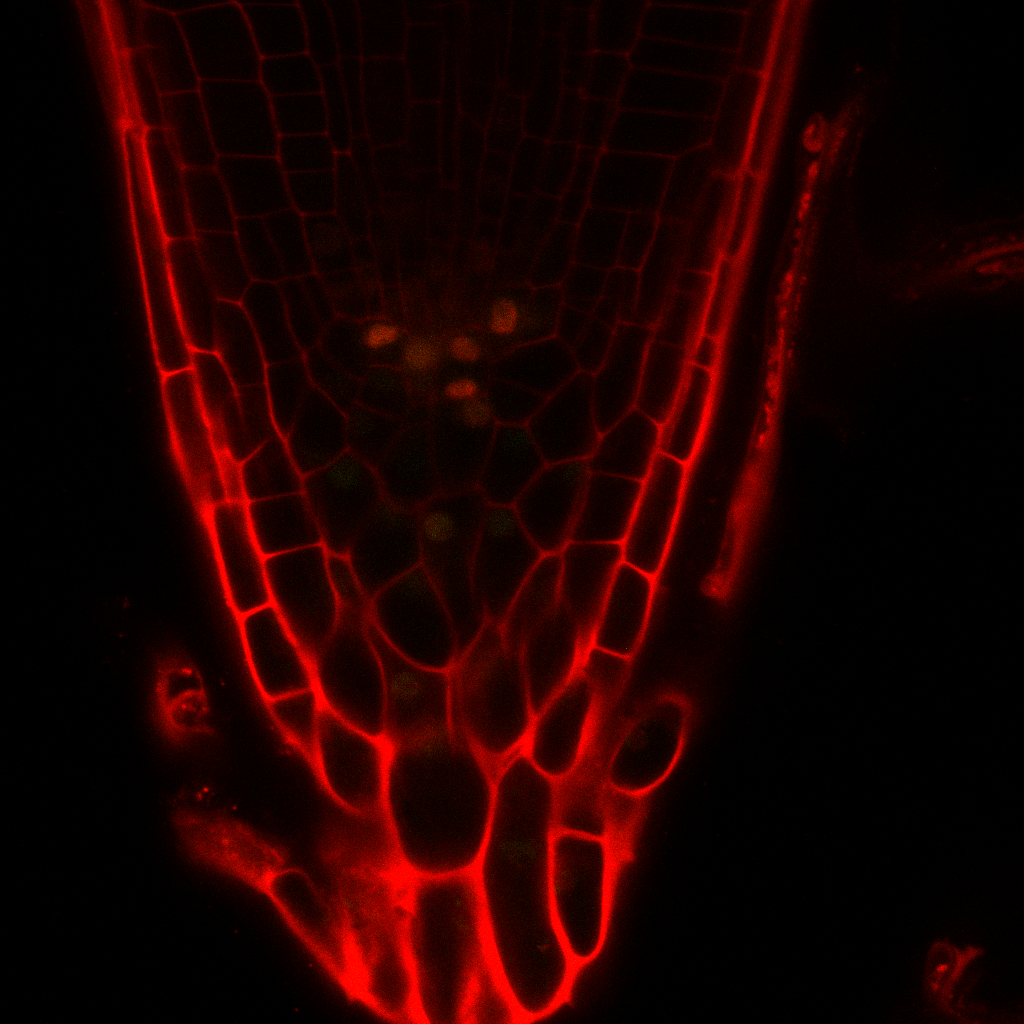

Supplement: Supplementary file 22 — Source data Fig. 5 [file 44318_2024_302_MOESM22_ESM.zip › Figure 5/5D/SGP1 in wox5-1_Image tdTomato and GFP.tif]

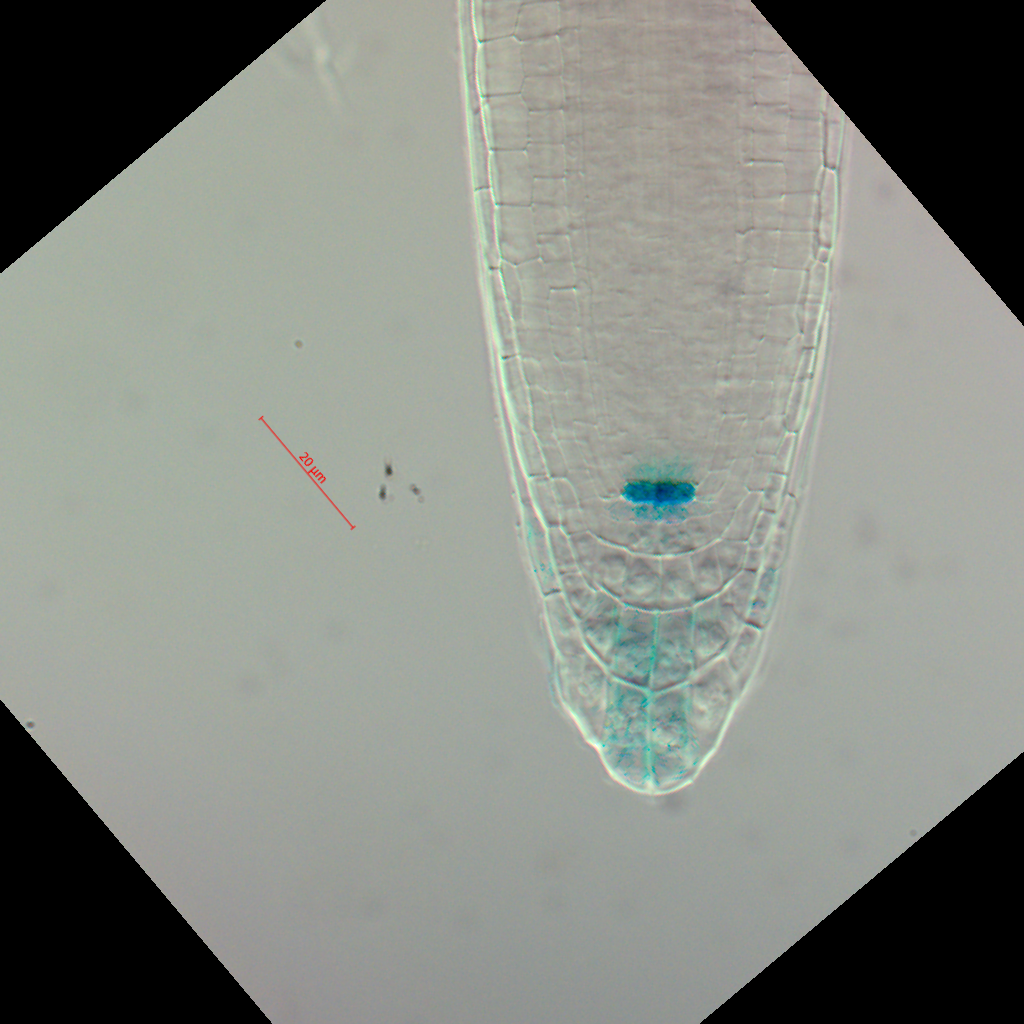

Supplement: Supplementary file 22 — Source data Fig. 5 [file 44318_2024_302_MOESM22_ESM.zip › Figure 5/5F/Figure 5F QC184.tif]

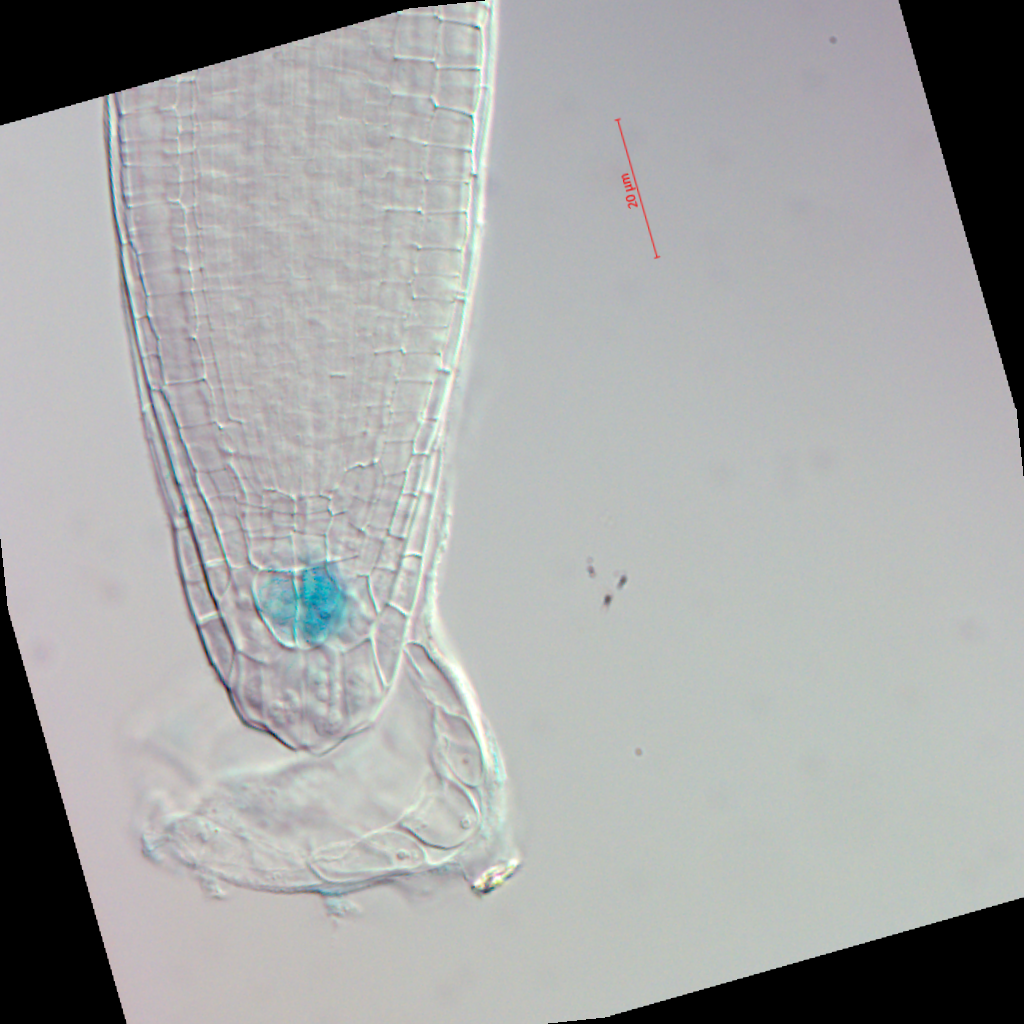

Supplement: Supplementary file 22 — Source data Fig. 5 [file 44318_2024_302_MOESM22_ESM.zip › Figure 5/5G/Figure 5G wox5-1 QC184.tif]

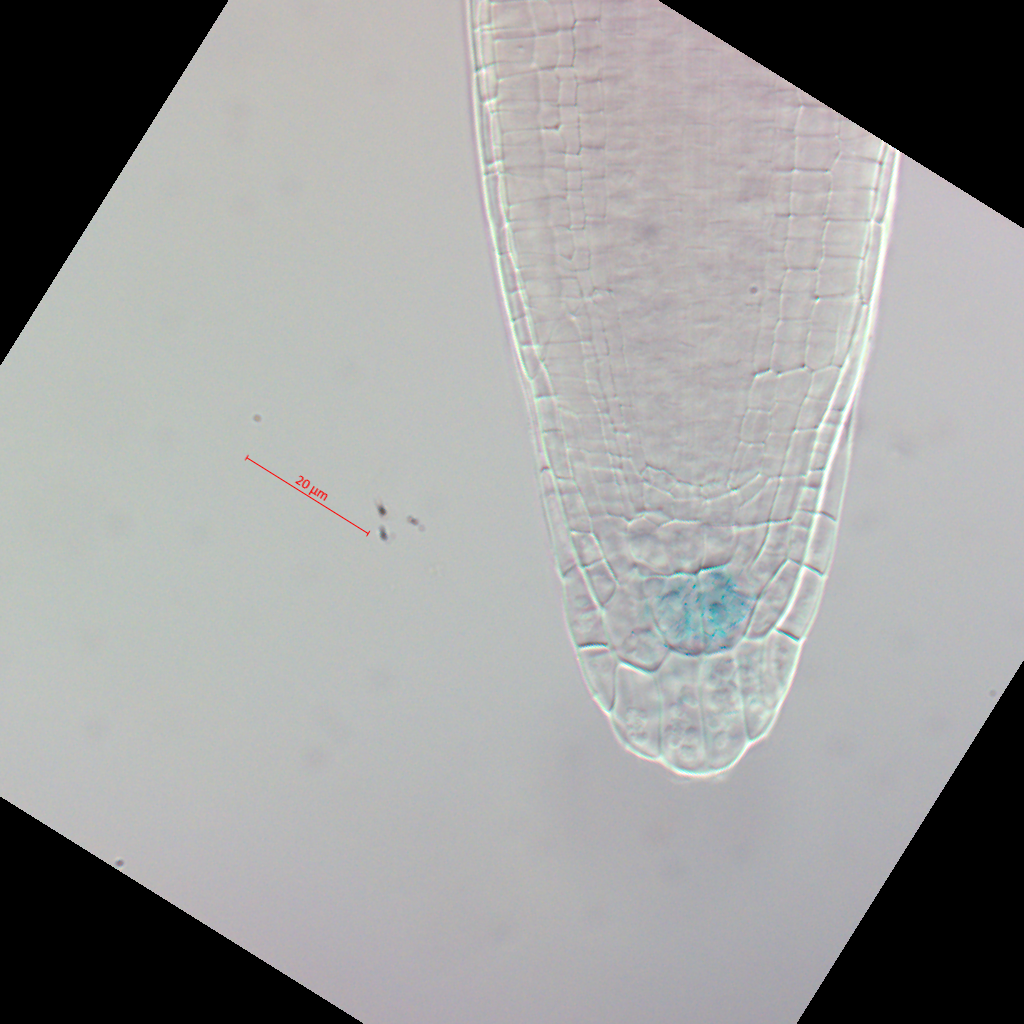

Supplement: Supplementary file 22 — Source data Fig. 5 [file 44318_2024_302_MOESM22_ESM.zip › Figure 5/5H/Figure 5H CESA1.tif]

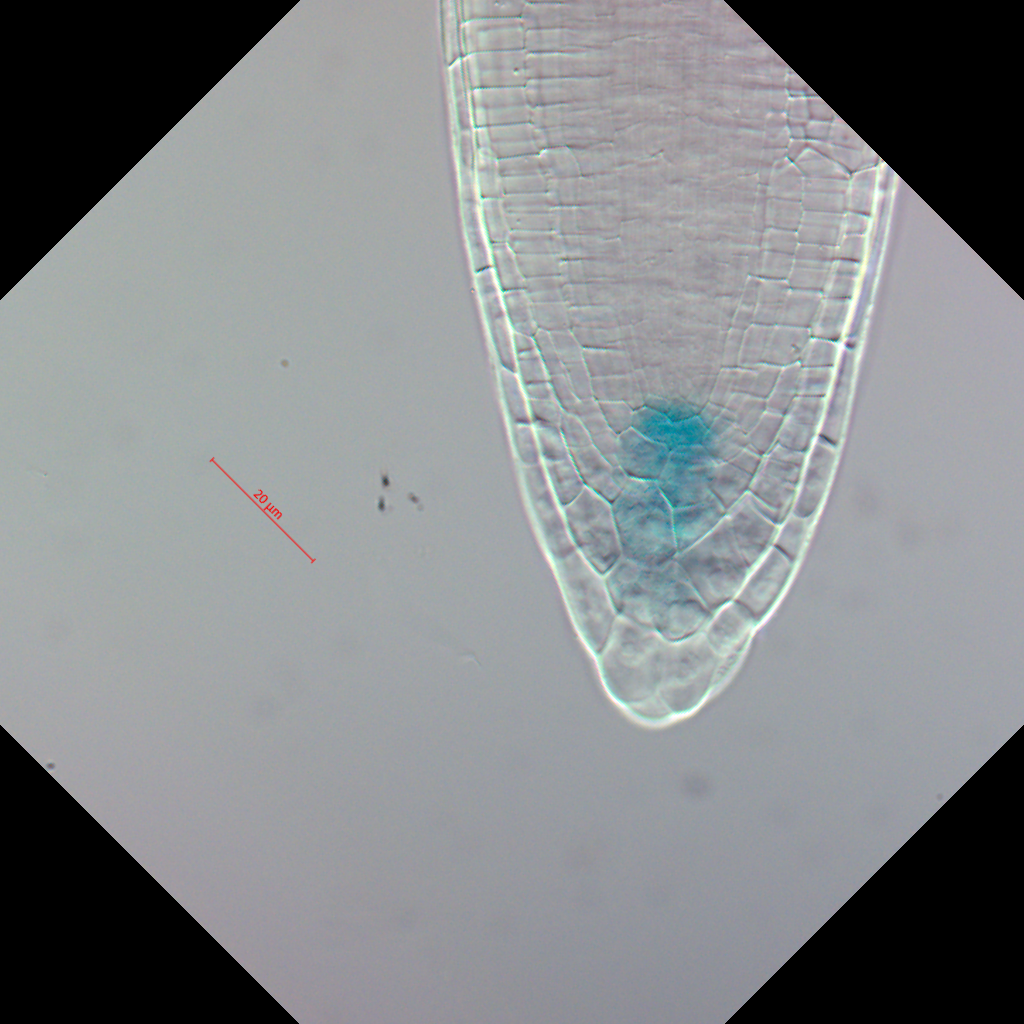

Supplement: Supplementary file 22 — Source data Fig. 5 [file 44318_2024_302_MOESM22_ESM.zip › Figure 5/5I/Figure 5I CEPR2.tif]

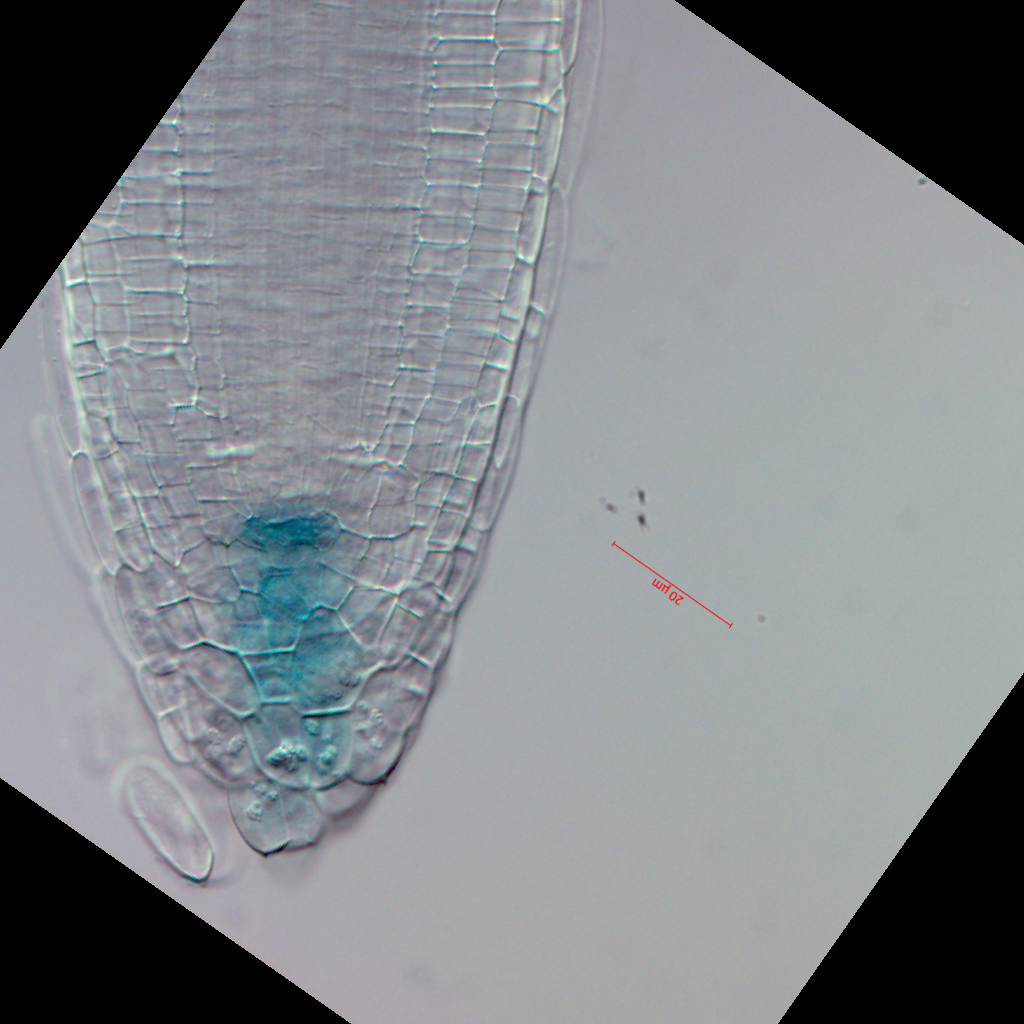

Supplement: Supplementary file 22 — Source data Fig. 5 [file 44318_2024_302_MOESM22_ESM.zip › Figure 5/5J/Figure 5J ERD14.tif]

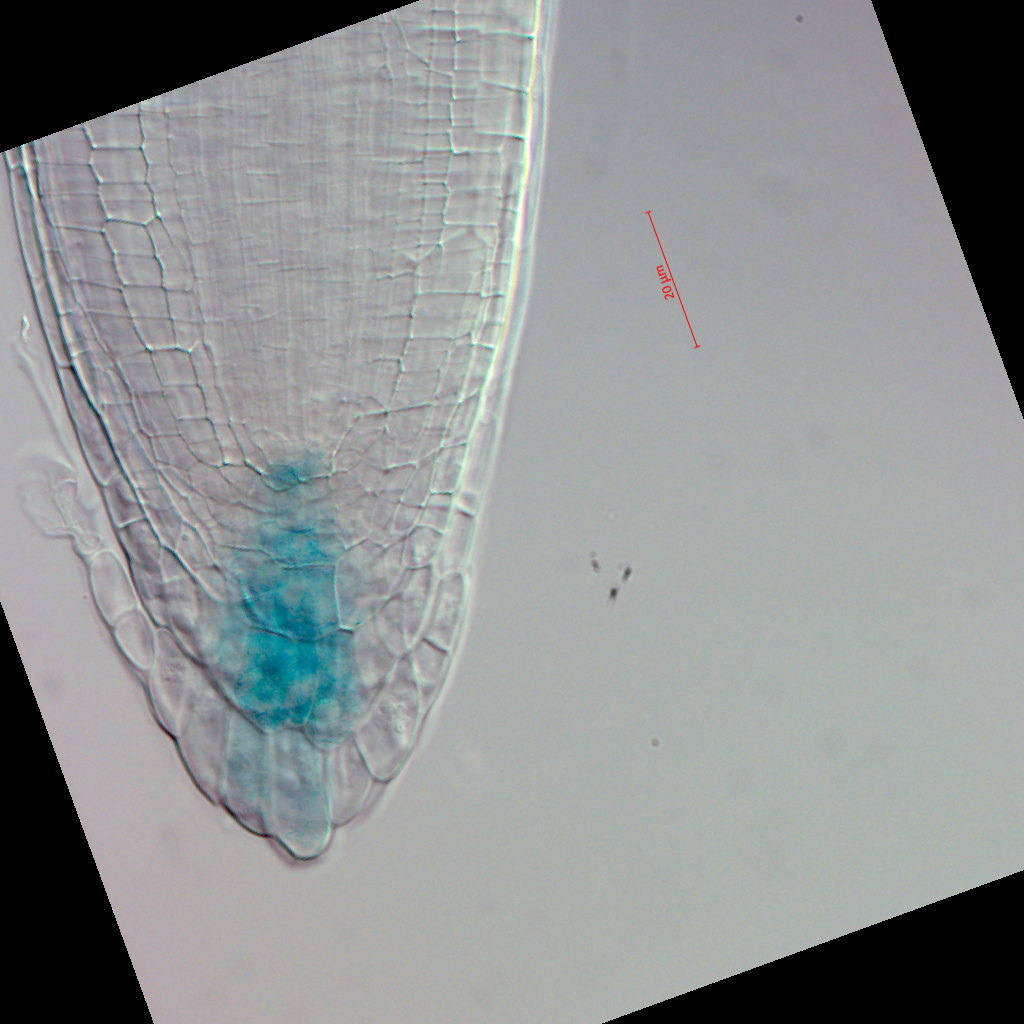

Supplement: Supplementary file 22 — Source data Fig. 5 [file 44318_2024_302_MOESM22_ESM.zip › Figure 5/5K/Figure 5K SGP1.tif]
