# Supplementary material for: Deciphering the molecular logic of WOX5 function in the root stem cell organizer
Source: EMBO J. 2024 Nov 18;44(1):281–303. doi: 10.1038/s44318-024-00302-2 (PMC11696986; doi:10.1038/s44318-024-00302-2)
Supplement: Supplementary file 23 — Expanded View Figures [file 44318_2024_302_MOESM23_ESM.pdf]

Expanded View Figures

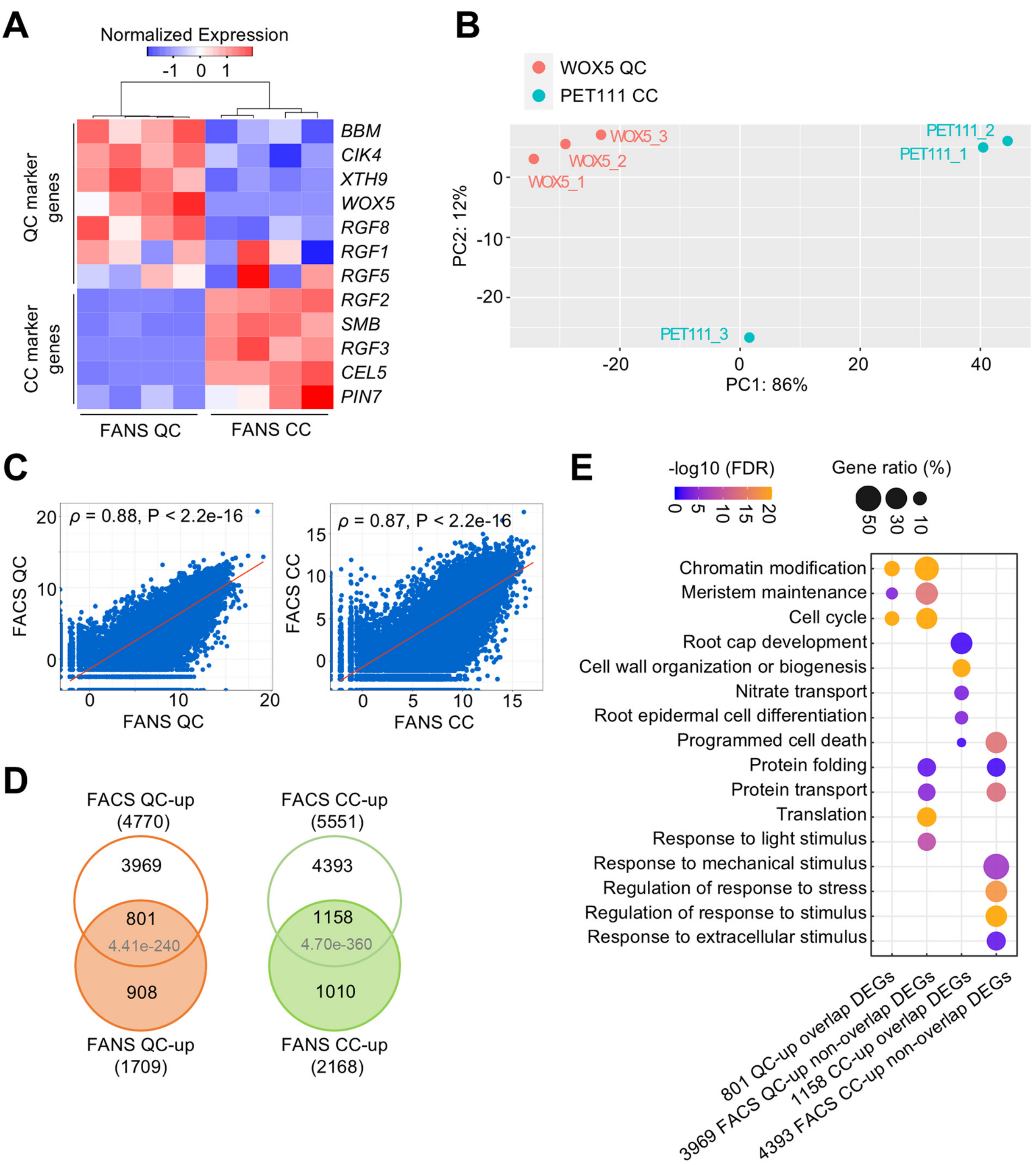

Figure EV1. Comparing FACS and FANS data of CC and QC.

(**A**) Heatmap of the expression values of published QC- and CC-associated genes in the QC-up and CC-up FANS transcriptome data. Four biological replicates are compared. The hierarchical clustering of the samples is based on Euclidean distance. (**B**) PCA of published FACS transcriptomes of QC and CCs (Li et al, 2016). Each dot represents one biological replicate. The first two components with the largest contributions are shown. Replicate PET111_3 was considered an outlier and removed for further analysis. (**C**) Pairwise correlation of QC and CC transcriptomes from this study (FANS) and from published sorted protoplast data (FACS, Li et al, 2016). The red line represents a linear regression. Spearman's rank correlation coefficients (ρ) and P values are shown. Correlation P value between FACS QC and FANS QC is < 2.2e-16. Correlation P value between FACS CC and FANS CC is < 2.2e-16. (**D**) Venn diagrams of FANS DEGs (CC/QC) from this study and FACS DEGs (CC/QC) from (Li et al, 2016). The P values indicate the statistical significances of the overlaps by hypergeometric tests. (**E**) GO analysis of shared and non-shared DEGs from FANS (this study) and FACS (Li et al, 2016) transcriptomes as shown in (**C**). The color gradient indicates –log10 (FDR) by Fisher exact test and Yekutieli correction (FDR < 0.05).

© The Author(s)

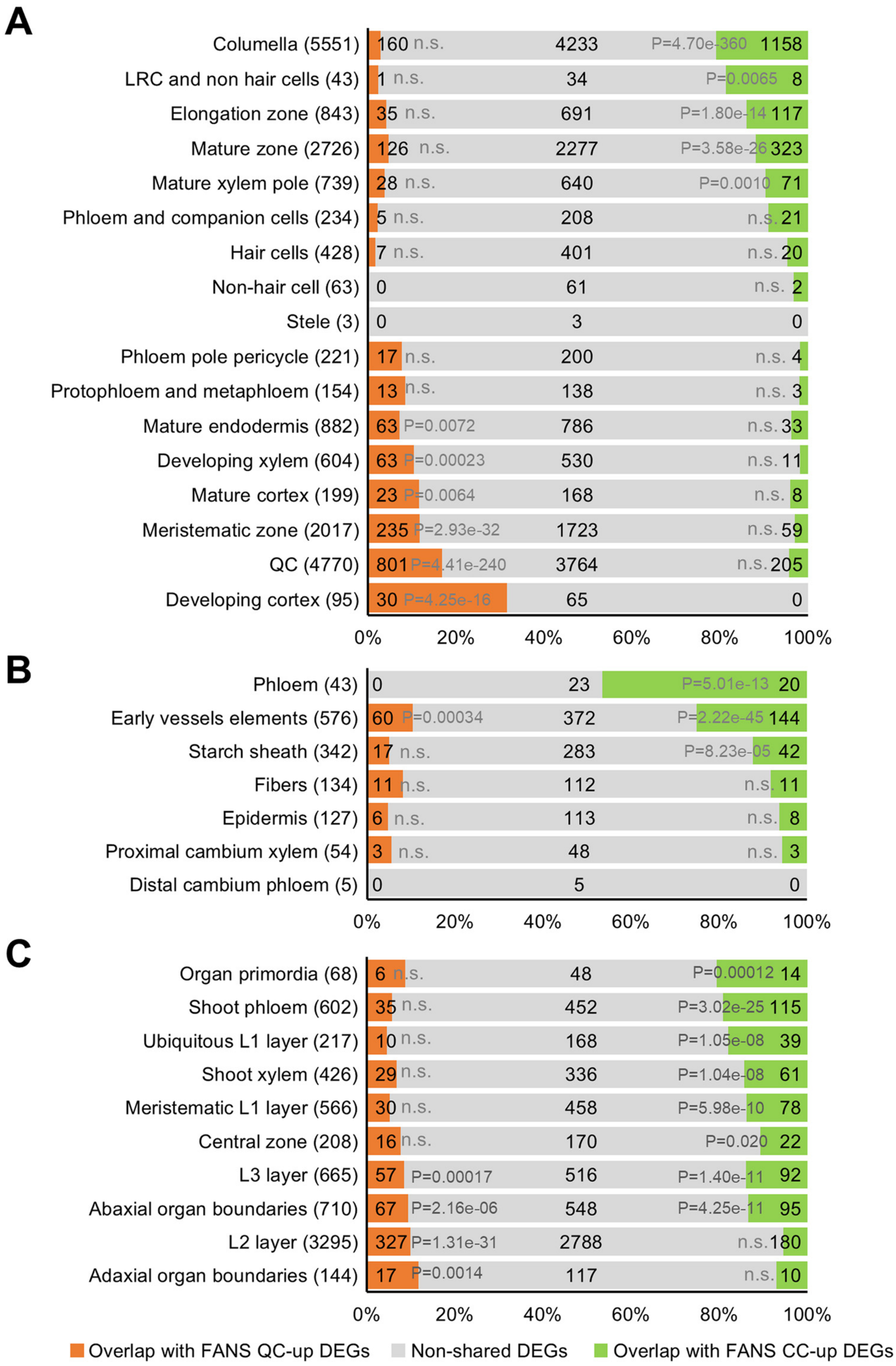

Figure EV2. Comparison of FANS CC and QC DEGs from this study with published data of different tissues.

(**A–C**) Comparison of FANS-derived DEGs from this study with tissue-specific DEGs from FACS of root cells (Li et al, 2016) (**A**), FANS of vascular cells (Shi et al, 2021) (**B**) and FACS of shoot apical meristem cells regions (Yadav et al, 2014) (**C**). The numbers of the published tissue-specific DEGs are shown in brackets. The numbers of shared DEGs and non-shared DEGs are indicated. *P* values by hypergeometric test are shown; n.s., not significant.

 © The Author(s)

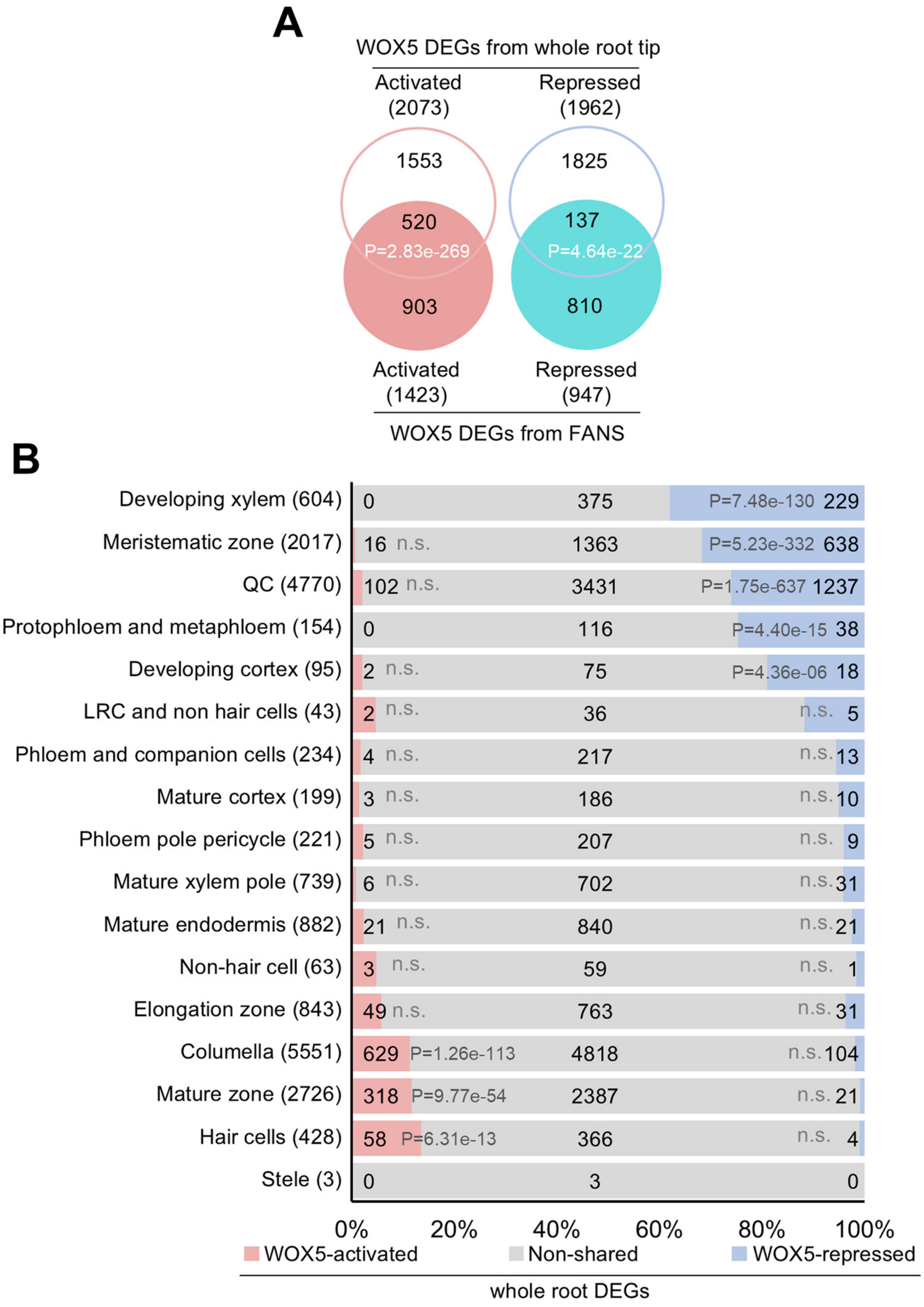

Figure EV3. Comparison of WOX5 DEGs from sorted nuclei and the whole-root tip.

(**A**) Venn diagrams of WOX5 DEGs from FANS transcriptomes (this study) and whole-root-tip transcriptome (*P*adj <0.05, FC ± 1.5, Clark et al, 2020). *P* values indicate the statistical significance of the overlaps by hypergeometric tests. (**B**) Comparison of the WOX5 DEGs derived from whole-root tips (Clark et al, 2020), which are not shared with our FANS data, with FACS-derived DEGs of different root regions from (Li et al, 2016). The numbers of the published tissue-specific DEGs are shown in brackets. The numbers of shared DEGs and non-shared DEGs are indicated. *P* values by hypergeometric test are shown; n.s., not significant.

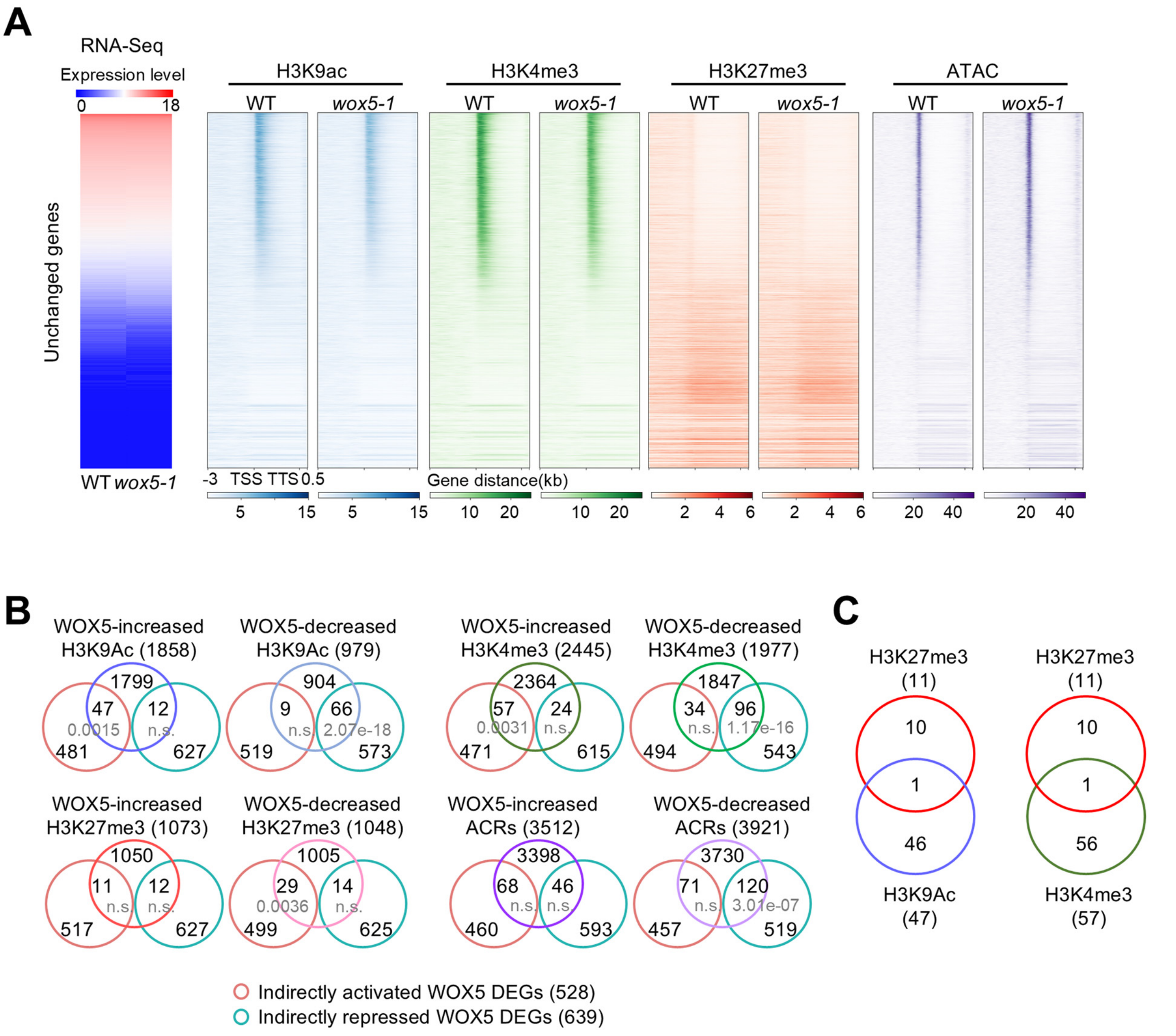

Figure EV4. Integrated analysis of indirect WOX5 DEGs, dHMs, and dACRs between *wox5-1* and WT QC.

(**A**) Histone modification and chromatin accessibility profiles between 3 kb upstream of TSS and downstream 0.5 kb of TTS of unchanged genes (unchanged expression between *wox5-1* and wild-type QC). (**B**) Venn diagrams of indirectly regulated WOX5 DEGs and the genes assigned to dHMs and dACRs. *P* values indicate the statistical significance of the overlaps by hypergeometric tests. (**C**) Comparison of WOX5-increased activating and repressive dHMs reveals no enrichment of bivalent marks in indirect regulated WOX5 DEGs.

 © The Author(s)

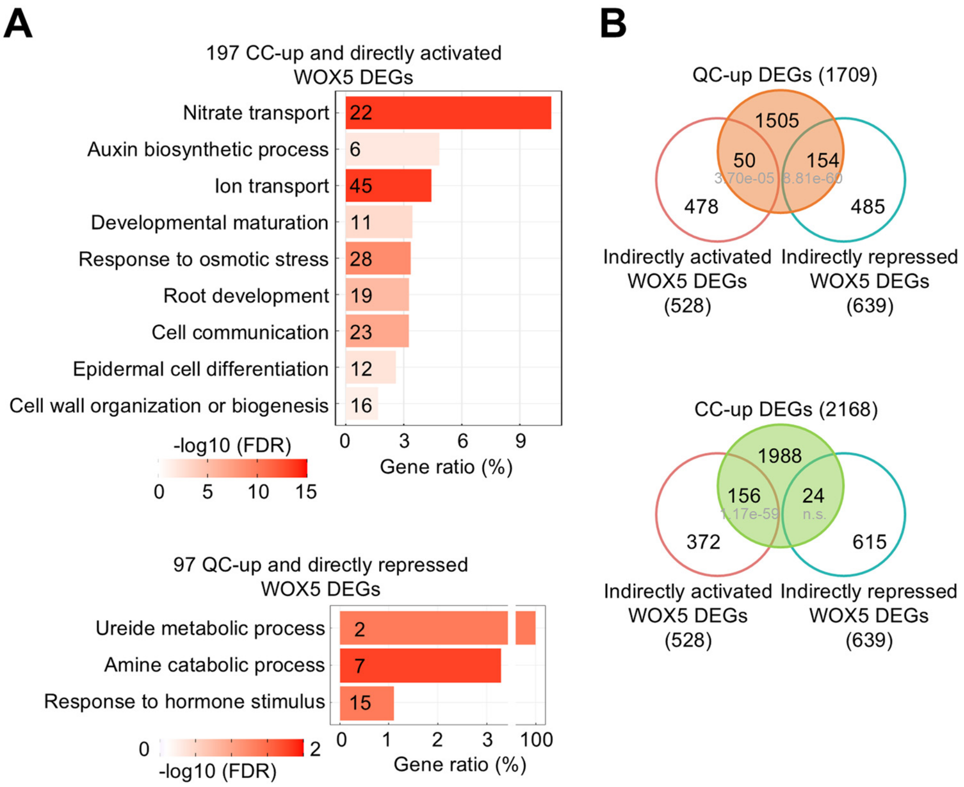

Figure EV5. Integrated analysis of QC-up/CC-up DEGs and WOX5 DEGs.

(**A**) GO analysis of the "paradoxical" gene subset. The color gradient indicates –log10 (FDR) by Fisher exact test and Yekutieli correction (FDR < 0.05). The number of genes in each term is indicated. (**B**) Venn diagrams of QC-up/CC-up DEGs and indirectly regulated WOX5 DEGs. *P* values indicate the statistical significance of the overlaps by hypergeometric tests.

© The Author(s)
